# Uterine Microbiota and Bisphenols: Novel Influencers in Reproductive Health

**DOI:** 10.3390/jox15010026

**Published:** 2025-02-02

**Authors:** Dafne Castellanos-Ruiz, J. Gerardo Ojeda-Borbolla, Olga V. Ruiz-García, Sheila I. Peña-Corona, Annia A. Martínez-Peña, María Elena Ibarra-Rubio, Marina Gavilanes-Ruiz, C. Adriana Mendoza-Rodríguez

**Affiliations:** 1 Facultad de Química, Departamento de Biología, Universidad Nacional Autónoma de México, Ciudad de México 04510, Mexicomeir@unam.mx (M.E.I.-R.); 2 Facultad de Química, Departamento de Farmacia, Universidad Nacional Autónoma de México, Ciudad de México 04510, Mexico; 3 División de Ciencias de la Salud, Universidad Intercontinental, A. C., Ciudad de México 14420, Mexico; 4 Facultad de Química, Departamento de Bioquímica, Universidad Nacional Autónoma de México, Ciudad de México 04510, Mexico

**Keywords:** microbiota, bisphenols, uterus, fertility, tight junctions, Toll-like receptors

## Abstract

Infertility affects 8–12% of couples worldwide, and 30–75% of preclinical pregnancy losses are due to a failure during the implantation process. Exposure to endocrine disruptors, like bisphenols, among others, has been associated with the increase in infertility observed in the past decades. An increase in infertility has correlated with exposure to endocrine disruptors like bisphenols. The uterus harbors its own microbiota, and changes in this microbiota have been linked to several gynecological conditions, including reproductive failure. There are no studies on the effects of bisphenols on the uterine–microbiota composition, but some inferences can be gleaned by looking at the gut. Bisphenols can alter the gut microbiota, and the molecular mechanism by which gut microbiota regulates intestinal permeability involves Toll-like receptors (TLRs) and tight junction (TJ) proteins. TJs participate in embryo implantation in the uterus, but bisphenol exposure disrupts the expression and localization of TJ proteins. The aim of this review is to summarize the current knowledge on the microbiota of the female reproductive tract (FRT), its association with different reproductive diseases—particularly reproductive failure—the effects of bisphenols on microbiota composition and reproductive health, and the molecular mechanisms regulating uterine–microbiota interactions crucial for embryo implantation. This review also highlights existing knowledge gaps and outlines research needs for future risk assessments regarding the effects of bisphenols on reproduction.

## 1. Introduction

Environmental pollution, as a consequence of urbanization and industrialization, has raised the incidence of health issues caused by endocrine disruptors (EDs), which are xenobiotics that can interact with hormone receptors, altering the endocrine system in the exposed individual. One of the most studied EDs is bisphenol A (BPA), a compound used as an important intermediate in the production of epoxy resins and polymers. This ED is used to provide desirable properties to a wide range of products, including bottles, liners, pipes, dental sealants, food packaging, children’s toys, nail polish, fire retardant materials, medical and electronic equipment, thermal paper, etc. [1]. As a result, humans are continuously exposed to this ED, which has been detected in urine, amniotic fluid, blood of adults and neonates, placenta, umbilical cord blood, and human breast milk at a range of levels known to be biologically active (>10 μg/L) [2].

**Figure 1:**
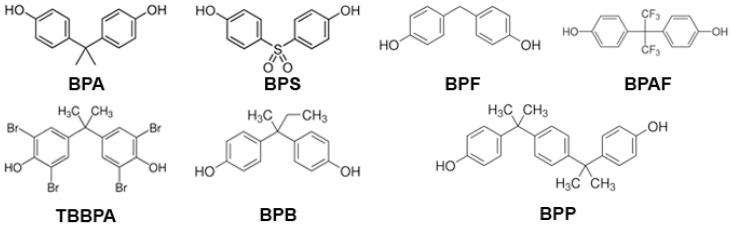
Chemical structure of bisphenols: BPA, bisphenol A; BPAF, bisphenol AF; BPB, bisphenol B; BPF, bisphenol F; BPP, bisphenol P; BPS, bisphenol S; and TBBPA, tetrabromobisphenol A.

Due to growing concerns about the detrimental effects of BPA on reproductive and metabolic health, its use was banned in the manufacturing of baby bottles, sippy cups, and infant formula packaging in the European Union (EU), the United States, and Canada [3]. In Denmark, Belgium, Sweden, and France, more bans have been established on the use of BPA in food contact materials and coatings [4]. Consequently, BPA analogs, like bisphenol S (BPS), bisphenol F (BPF), bisphenol AF (BPAF), and tetrabromobisphenol A (TBBPA) (Figure 1), have gradually replaced BPA in many consumer products labeled as “BPA-free”, resulting in a significant increase in human exposure to these substances. Unfortunately, little or no research was conducted to determine the safety of these BPA-free products before they were marketed to the public as a healthier alternative. These analogs are used in the production of everyday use products. BPS is used in paper currency and cashier’s receipts; BPF in tank and pipe linings, dental sealants, and food packaging materials; BPAF in the plastics industry; and TBBPA is applied in plastics, paper, textiles, and circuit boards as a flame retardant. As a consequence of their unrestricted use, all these BPA substitutes have been detected in human samples around the world: BPF, BPAF, and BPS were detected in urine samples with a concentration of 0.15 to 0.54 μg/L, below the detection level to 3.93 μg/L, and 0.654 ng/mL (which was comparable to BPA), respectively. Meanwhile, TBBPA was identified in human serum and breast milk samples at levels of 480 ng/L and between 50 and 350 pg/kg/day, respectively [5,6,7,8,9,10,11]. BPA and its chemical structure analogs are similar to steroid hormones, so they can bind to membrane and nuclear receptors, such as estrogen, androgen, and thyroid hormone receptors. In doing so, they may produce endocrine disruption, tumors, adverse reproductive outcomes, and transgenerational effects, posing a threat to human health [12].

Microbiota, the diverse community of commensal, symbiotic, and pathogenic microorganisms that colonize different parts of the body of all animals [13,14], plays a key role in the host’s health and disease by regulating many diverse and complex biological processes such as brain development and behavior, metabolism, immune response, and reproduction [15,16,17]. Having over 3 million unique genes, the human microbiota is often referred to as a “second genome”. Its composition can vary or be disrupted due to various intrinsic and extrinsic factors, including host genetics, body location, diet, and exposure to xenobiotics [17,18]. Indeed, bisphenols (mainly BPA, BPS, and BPF) can accumulate at bacterial membranes due to their lipophilicity, disrupting and disturbing membrane permeability and cell function and unchaining cell destruction [19,20]. Importantly, bisphenols have also been shown to alter microbial composition in both soil and gut microbiomes [13,21]. Research has shown that microbiota and xenobiotics, including endocrine disruptors like bisphenols, may potentially modulate adverse health effects via microbial-xenobiotic interactions [22].

For nearly a century, it was believed that the uterus was a sterile environment, with any kind of colonization of the upper reproductive tract linked solely to infections, diseases, or health problems. However, in 2008, the Human Microbiome Project revealed the presence of microbiota in body sites previously considered sterile, such as the female upper reproductive tract [23]. Since then, several studies have attempted to establish a “baseline” or “core” microbiome of the healthy endometrium; however, due to limitations in these studies, this has not been fully achieved. Nevertheless, identifying microbial communities in the reproductive tract of healthy women and those with gynecological diseases has shown that microbial dysbiosis may be associated with different gynecological disorders and with treatment success in assisted reproductive technology (ART).

This review aims to summarize information about the microbiota in the female reproductive tract (FRT), its association with different reproductive diseases, especially reproduction failure, the impact of bisphenols on microbiota composition and reproduction, and the molecular mechanisms that may regulate interactions between the uterus and microbiota, which are important for embryo implantation. Additionally, we identify knowledge gaps and research needs for future risk assessments concerning the effects of bisphenols on reproduction.

## 2. The Cervicovaginal Microbiota Impacts on Reproduction

Given the crucial role of the microbiome in human physiology, humans have been described as holobionts or communities composed of the host and its symbiotic microbes rather than individuals [24]. Interestingly, the combination of the host genome and microbiome enhances genetic variation and phenotypic plasticity, allowing the holobiont to improve its overall fitness. Consequently, bacteria might arguably play an essential role in producing reproductively fit and healthy offspring in addition to influencing the overall health of an individual.

The FRT can be divided into two connected parts: the upper and lower reproductive tracts. The former includes the ovaries, fallopian tubes, and uterus, while the latter comprises the cervix and vagina.

The cervicovaginal microbiome impacts several important reproductive outcomes, including preterm birth, fertility, cervicitis, and risk of sexually transmitted infections (STIs) [25]. Twenty different genera of bacteria have been recognized in the vagina [26]. Although *Lactobacilli* (*L*.) are most often the dominant species in the vagina (99.97%), significant changes in the vaginal microbiota have been reported between individuals and even in the same person under different circumstances, like the follicular phase, the secretory phase, during menstruation, and sexual intercourse [27,28,29,30,31,32]. In the cervix, the microbiota composition is similar to the one in the vagina, although in a lower amount (vagina: 10^10^–10^11^; cervix: 10^7^–10^8^) [28,33]. In particular, it has been proposed that *L. crispatus* and *L. gasseri* species are related to the preservation of a simple vaginal microbiome, dominating their respective vaginal communities and providing a barrier against opportunistic pathogens. This can be explained by the production of bacteriostatic and bactericidal compounds (e.g., lactic acid and hydrogen peroxide) that preserve a low pH (≤4.5). Furthermore, *L. crispatus* is probably involved in a successful pregnancy [34,35]. Even though *L. iners* has been related to health-promoting effects, it is also able to increase the vaginal pH, producing microbiome perturbation and species-specific virulence factors [35]. Moreover, it has been reported that a *L. iners*-dominated cervicovaginal microbiota at gestation is associated with an increased risk of a short cervix or preterm birth [36,37].

However, not all women have *Lactobacillus*-dominant vaginal flora. Women with a low percentage of *Lactobacillus* in their vaginal sample are less likely to have a successful embryo implantation [38,39]. Preterm birth and a short cervix are also associated with a community characterized by a scarcity of *Lactobacillus* species and a wide array of anaerobic bacteria [40], which are also typically found in common vaginal infections, such as bacterial vaginosis, where the overgrowth of typically non-*Lactobacillus* anaerobic bacteria, including *Gardnerella vaginalis*, *Mobiluncus* spp., and *Atopobium vaginae*, leads to a disruption of the ecological vaginal balance [32]. Furthermore, for patients undergoing in vitro fertilization (IVF), *Lactobacillus*, *Akkermansia*, *Desulfovibrio*, *Atopobium*, *Prevotella, and Gardnerella* showed differential abundance between pregnant and non-pregnant women. Among them, *L. iners* was the predominant group in the non-pregnant IVF patients and was negatively correlated with the other genera and positively correlated with serum estradiol (E_2_) levels. Meanwhile, in the pregnant IVF patients, *L. crispatus* was the predominant group and vaginal progesterone (P_4_) did not appear to impact the vaginal microbiota during pregnancy [37,41,42].

While it is known that there is a connection between an optimal *Lactobacillus*-dominant microbiota and favorable reproductive health outcomes, the mechanisms through which this occurs are still unknown [43].

The mucosal surface of the FRT is the primary site of entry for many STIs, including HIV. It is composed of a non-keratinized stratified squamous epithelium that forms a substantial physical and immunological barrier against pathogens [44,45,46]. Interactions between the FRT epithelial cells are mediated by intercellular junctional molecules comprising tight junction (TJ) complexes, which are a hallmark of the vaginal mucosa. Under specific conditions, the permeability of these complexes is altered to permit passage of innate immune effector molecules secreted by the vaginal epithelial cells [45].

It has been reported that colonization of cell multilayer cultures from the vaginal epithelium by common vaginal commensals, including *L. crispatus*, *L. jensenii*, and *L. rhamnosus*, led to an intimate association of *Lactobacillus* with the epithelial cells. This exclusively occurs on the apical surface and protects it from *Staphylococcus epidermidis* colonization, which can trigger cytokine secretion and produce inflammation [45]. One mechanism by which cervicovaginal *Lactobacilli* improve the integrity of the FRT epithelial barrier may involve the metabolite lactic acid, which directly strengthens the barrier by upregulating the expression of TJ proteins claudin-1 and claudin-4 in vitro [43]. It has been demonstrated that *L. crispatus* and soluble factors from this species accelerated the re-epithelialization of vaginal epithelial cells and augmented vascular endometrial growth factor secretion [47].

## 3. Microbiota in the Uterus

Since 1900, the uterus was considered a sterile environment for microorganisms until the mid-1980s, when it was demonstrated that the cervical mucus plug was not entirely impermeable to bacterial ascension from the vagina [48]. Due to the challenges of simulating living organ conditions with culture methods, colonization in the upper reproductive tract has only been associated with infections, diseases, or health issues such as endometriosis or preterm birth [49,50,51]. Furthermore, several IVF clinical studies indicated that bacterial contamination of the embryo transfer catheter significantly reduces clinical pregnancy rates [52,53,54,55]. Thus, eradication of endocervical microorganisms with the administration of prophylactic antibiotics has been attempted to improve implantation [52,53]. In 2008, the Human Microbiome Project laid the groundwork for research on human microbiota, revealing the presence of microbiota in body sites once thought to be sterile, thanks to advances in sequencing techniques [23]. Short-read 16S rRNA sequencing-based analysis of microbiome profiling generally provides information on microbial compositions at the genus level.

Common applications include identifying microbiome composition across various sample groups to investigate microbial differences between groups and conditions. Chen et al. identified distinct microbial communities in the vagina, cervical canal, uterus, fallopian tube, and peritoneal fluid, demonstrating that the FRT is not sterile [28]. These findings challenged the dogma that a healthy uterine cavity was sterile and that the presence of microbes was a sign of pathology (ascension of bacteria through the cervix, through blood, or as a result of gynecological procedures like ART or insertion/removal of intrauterine devices). In addition, it was demonstrated that the endometrial microbiota (EM) detected was not due to contamination of the samples by vaginal microbiota since some bacteria genera present in the endometrium were absent in the vagina of the same subject, and vice versa [26]. The Human Microbiome Project has revealed that approximately 9% of the total human microbiome is found in the FRT [56,57].

Recent studies that analyzed the EM helped to identify microbial communities in healthy women, revealing that microbial dysbiosis could be associated with different gynecological disorders and with treatment success in ART [26,58]. The predominant phyla identified in the endometrium of healthy women were *Firmicutes* (mainly *Lactobacillus* spp.), *Bacteroidetes* (*mainly Flavobacterium* spp., *Bacteroides* spp., *Prevotella* spp.), *Proteobacteria* (mainly *Pseudomonas* spp., *Acinetobacter* spp.), and *Actinobacteria* (mainly *Gardnerella* spp., *Bifidobacterium* spp.) [24,26,48,59,60,61,62]. The endometrium possesses a higher bacterial diversity with a lower bacterial biomass as compared with those in the vagina (endometrium biomass: 10^6^–10^7^; vagina biomass: 10^10^–10^11^) [28].

The endometrium proliferates and dies during the menstrual cycle in order to provide an adequate environment for implantation and pregnancy. These changes are driven by variations in ovarian steroid hormones [26]. However, it is still unclear if the changes in ovarian steroid hormones induce alterations in the uterine microbiome during the menstrual cycle. Studies in endometrial samples showed a higher abundance of *Prevotella* spp. during the proliferative phase, while increased *Sneathia* spp. were observed during the secretory phase [28,63,64]. It was reported that some bacteria were exclusively detected in specific phases. Bacteria genera such as *Actinobaculum*, *Mobiluncus*, and *Porphyromonas* were only discovered in the proliferative phase, while other genera like *Aerococcus*, *Delftia*, and *Sneathia* were only identified in the secretory phase. Moreover, predominant species were different between phases as well. The most frequent bacteria genera in both phases were *Bifidobacterium*, *Burkholderia*, *Gardnerella*, *and Lactobacillus.* Meanwhile, *Escherichia and Prevotella* were the most common genera in the proliferative phase, while *Atopobium* and *Streptococcus* were in the secretory phase [28,64,65]. Low levels of *Lactobacillus* were detected after menstruation, and they gradually increased during the proliferative phase, with a peak during the secretory phase [66]. In contrast, other investigations showed stable microbiota profiles in >80% of patients analyzed during two different time points of the secretory phase of the menstrual cycle (LH+2 and LH+7), suggesting that the EM is not hormonally regulated during the acquisition of endometrial receptivity [26]. Moreover, the EM may be altered by exogenous hormones, including those used for ovarian stimulation, progesterone (P_4_) supplementation, and various types of ovulation induction and luteal support used during IVF [63]. For instance, progestin administration has been associated with a loss of *Lactobacillus* spp. diversity and the emergence of *L. crispatus* as the dominant phylotype in the endometrium of women with menorrhagia and dysmenorrhea [64]. Additionally, ovarian stimulation and P_4_ luteal supplementation were associated with a slight decrease in the proportion of *Lactobacilli* and an increase in *Prevotella* and *Atopobium* in the endometrium of women undergoing IVF [67].

While it is still unclear how the uterus is colonized, the ascension of bacteria from the vagina is one of the most possible paths [23,51]. Similar to vaginal microbiota, the endometrium is mainly dominated by *Lactobacillus* spp. and its depletion is associated with preterm birth and infertility too [24]. However, despite the similarities, it has been observed that bacterial composition in the vagina greatly differs from the intrauterine microbiome [48,68]. The divergence in microbiomes could be due to the differences between the tissues since the upper reproductive tract is lined by a monolayer of columnar epithelial cells while the vagina is lined by a layer of non-keratinized squamous epithelium and has a lower pH, which would produce a different environment for the growth of microorganisms [69,70]. Others suggested that there may be other routes of uterus colonization, like the hematogenous transfer of microbiota from another site, such as the gut or oral microbiota to the uterine cavity; the transfer of microbes through the fallopian tubes; via insertion of intrauterine devices; the possible transportation of microbiota in the external environment or the lower genital tract into the uterine cavity by sperm (spread with sperm); and gynecological procedures related to ART [51,71].

The functions of the EM include: (a) participation in the proliferation and apoptosis of endometrial cells; (b) improving the anti-infection capacity of the endometrium by preventing the proliferation and attachment of pathogenic microorganisms to the endometrial surface; (c) regulation of the uterine immune response as a result of the production of inflammatory cytokines, chemokines and antibacterial substances which are induced by the binding of microbial ligands to host receptors; and (d) participation in blastocyst implantation and pregnancy maintenance [71].

## 4. Uterine Microbiota in Gynecological Diseases

Studies on EM in gynecological diseases have shown that women with chronic endometritis (CE) (persistent inflammation of the endometrial lining, characterized by the presence of edema, increased stromal cell density, and dissociated maturation of the stroma and epithelium throughout the menstrual cycle) had microbiomes with significantly higher proportions of *Firmicutes* and lower proportions of *Proteobacteria* than healthy women [68]. Moreover, it has been reported that the abundance of *Acinetobacter*, *Actinobacteria*, *Anaerococcus*, *Bifidobacterium*, *Dialister*, *Enterobacteriaceae*, *Enterococcus*, *Fusobacteria*, *Gardnerella*, *Klebsiella pneumoniae*, *Neisseria*, *Phyllobacterium*, *Prevotella*, *Sphingomonas*, *Staphylococcus*, and *Streptococcus* was significantly increased in chronic endometritis patients [72]. These bacteria may regulate an increase in immune cells, producing a decrease in the receptivity of the endometrium. Thus, this disease has been related to pregnancy failure of both spontaneous and ART conceptions since 14–41% of CE patients presented recurrent implantation failure (RIF), and 8–28% had repeated pregnancy loss (RPL) [73,74]. Infertile patients with CE who underwent IVF had a significantly lower clinical pregnancy rate (32%) as compared to infertile non-chronic endometritis patients (59.4%) [68,73,74,75,76,77]. The prevalence of CE has been estimated at 2.8–39% in infertile patients but can be as high as 60% or 66% in patients with inexplicable RPL or RIF, respectively [78].

In women with endometriosis (growth of endometrial tissues (glands and stroma) outside the uterine cavity), *Lactobacillacae* were significantly decreased, while *Staphylococcus*, *Streptococcaceae*, *Gardnerella*, *Enterococcus*, *Alishewanella*, *Prevotella*, *Acinetobacter*, *Vagococcus*, *Comamonas*, *Escherichia coli*, *Pseudomonas*, and *Moraxellaceae* were significantly increased, as compared to healthy women [79,80,81,82,83]. It has been proposed that the EM contributes to the incidence and progression of endometriosis by regulating the immune system [71].

In women with adenomyosis (presence in the myometrium of endometrial tissue), the endometrium was enriched by *Comamonadaceae*, *Enterobacteriaceae*, *Prevotella copri*, *Citrobacter freundii*, *Weissella confusa*, *Burkholderia cepacia*, *Lactobacillus zeae*, *Delftia* spp., *Acinetobacter* spp., *Shewanella* spp., *Peptoniphilus* spp., *Pseudomonas viridiflava*, *Tissierellaceae* (1–68 spp.), *Pseudomonas* spp. and *Corynebacterium* spp., as compared to control women [28,84].

The endometrium of women with hysteromyoma (benign tumors in the uterus) was enriched with *Ruminococcaceae*, *Alcaligenaceae,* and *Blastomonas natatoria* as compared to healthy women [28].

In patients with endometrial hyperplasia (increased gland-to-stroma ratio in the endometrium compared with the normal proliferative endometrium), an increase in the relative quantity of *Actinobacteria*, *Bacteroides*, *E.coli*, *Firmicutes*, *Fusobacteria*, and *Proteobacteria* has been reported, while *Lactobacillus* abundance was reduced [71,85]. The increase in these microorganisms may be related to the elevation in estrogen levels and the production of inflammatory cytokines (IL-6, IL-1β, and TNF-α) observed in these patients, factors that are implicated in the development, promotion, and progression of this pathology [85].

Several investigations have proposed that a dysfunction of the immune system, as well as an imbalance of the genital tract microbiota, may be involved in the incidence, development, and metastasis of gynecological malignancies. It has been reported that in endometrial cancer patients, the proportion of *Anaerococcus*, *Anaerostipes*, *Anaerotruncus*, *Arthrospira*, *Atopobium vaginae*, *Bacillus cereus*, *Bacillus pseudofirmus*, *Bacteroides*, *Bacteroides fragilis*, *Clostridium botulinum*, *Dialister*, *Micrococcus*, *Muribaculum*, *Mycoplasma hyopneumoniae*, *Nocardioides*, *Pasteurella multocida*, *Pelomonas*, *Peptoniphilus*, *Porphyromonas* spp., *Prevotella*, *Pseudomonas uter*, *Ruminococcus*, *Treponema*, and *Stenotrophomonas rhizophila* was higher than in healthy people, while the abundance of *Lactobacillus* and *Oscillibacter* was decreased [86,87,88,89,90,91,92,93].

Therefore, the microbiota components change in the different types of endometrial diseases. However, it is important to note that there are common bacteria in these disorders: *Bifidobacterium*, *Bacteroides*, *E. coli*, *Gardnerella*, *Lactobacillus*, *Prevotella*, *Pseudomonas*, *Staphylococcus*, and *Streptococcus*. In endometrial diseases, the proportion of *Lactobacillus* and *Firmicutes* declined, while the abundance of *Actinobacteria* (like *Gardnerella* and *Bifidobacteria*), *Bacteroidetes* (like *Bacteroides fragilis*, *Prevotella*, and *Bacteroides*), and *Proteobacteria* (like *Staphylococcus* and *E.coli*) augmented, as compared with healthy endometria. Thus, the changes in EM composition play a key role in endometrial pathology, making the microbiota a candidate to become a target for the prevention and treatment of various endometrial illnesses [71].

## 5. Uterine Microbiota and Reproduction

Infertility affects about 8–12% of couples worldwide and remains a major global concern both socially and economically. Its prevalence has increased in recent decades [94]. In couples afflicted by infertility, 26–30% of cases are produced by male factors, while 45–60% are generated by female factors [95]. While the underlying origin of human infertility has been potentially challenged by the successful utilization of IVF and embryo transfer techniques, the success rate of implantation remains frustratingly low. This is considered primarily due to the poor understanding of uterine receptivity during embryo transfer. Other factors that may impact the embryo–endometrium coordination, which affects implantation, are the maternal immune system, the reproductive tract microbiome, anatomical issues, hematological aspects, the endocrine environment, and embryo and parental genetics. Recurrent implantation failure (RIF) is described as the incapability to accomplish a clinical pregnancy after the transfer of at least four good-quality embryos in no less than three fresh or frozen cycles in a woman under 40 years of age [96]. In order to have a successful implantation, there has to be correct timing between the development of high-quality embryos and a receptive endometrium. Sixty-six percent of implantation failures are attributed to inadequate endometrial receptivity, while embryo quality is only responsible for 33% [97]. Therefore, it is essential to resolve the complex interplay between embryo development and endometrium receptivity to optimize success. Recent studies have indicated that the microbiota may participate in the interaction between hormones, immune cells, and physiological adaptations required for a favorable pregnancy [98]. Microbial dysbiosis, which is a change in the conformation, allocation, or functioning of the normal microbiota [96], may play a role in IVF failure, and the restoration of an adequate uterine microbiota could make available new clinical treatments for infertile couples [74]. However, although microbiota dysbiosis in the uterus appears to be related to unsuccessful implantation and birth, it is not yet clear whether it is a determining factor due to the limitations of the studies. In particular, hormonal and physiological changes within the menstrual cycle, the lack of ethnic diversity, differences in socioeconomic status and lifestyle, environmental factors and the challenge of collecting uncontaminated uterine samples, lacking vaginal or cervical bacteria and bacterial DNA found in the air and in laboratory reagents and equipment, have hindered progress in the identification of a “baseline” or “core” microbiome of the healthy endometrium [58,99]. However, in order to avoid contamination from vagina and cervical canal microbiota, some studies have employed a double-lumen catheter to obtain endometrial microbiota samples [62,72,96,100,101].

During early pregnancy, it has been described that *Lactobacillus*, the most common microbe identified in the endometrium, was associated with defense mechanisms in the reproductive tract, such as maintaining the pH balance, preventing prolonged colonization by harmful bacteria through adhesion to epithelial cells, producing lactic acid, hydrogen peroxide, and bacteriocins, and regulating the local immune system (Table 1) [102,103]. *Proteobacteria*, *Cupriavidus*, *Finegoldia*, *Microbacterium*, *Achromobacter*, and *Tepidimonas* are also beneficial bacteria since it has been reported that their relative abundance was significantly higher in successful pregnancy groups [72,74,104]. Studies in women undergoing IVF have suggested that a non-*Lactobacillus*-dominated EM (NLDM) (<90% *Lactobacilli* with >10% of other bacteria) was associated with infertility, with a significantly decreased implantation (NLDM 23.1% vs. *Lactobacillus*-dominated EM (LDM) 60.7%), significantly decreased pregnancy (NLDM 33.3% vs. LDM 70.6%), significantly decreased ongoing pregnancy (NLDM 13.3% vs. LDM 58.8%), and significantly decreased live births (NLDM 6.7% vs. LDM 58.8%) (Table 1) [26,62,96,100,101,102]. Another study suggested that the amount, rather than the proportion, of *Lactobacillus* in the endometrium plays a role in developing endometrial receptivity, as patients with extremely low microbial biomass were strongly linked to a pre-receptive endometrium (Table 1) [105]. In contrast, other authors reported that endometrial bacterial colonization was found to consist of a polymicrobial environment, with *Lactobacilli* being uniquely present in the group that experienced unsuccessful IVF outcomes. This microbiota may originate from the vagina due to a failure of the barriers that typically block such migration (Table 1) [106]. Moreover, other IVF studies indicated that the pregnancy, implantation, and miscarriage rates were comparable between infertile patients with an eubiotic endometrium (≥80% *Lactobacillus* + *Bifidobacterium* spp.) and dysbiotic endometrium (<80% *Lactobacillus* + *Bifidobacterium* spp. with ≥20% of other bacteria) (Table 1) [101,107]. Furthermore, some patients achieved pregnancies despite having 0% *Lactobacillus* and 95.5% *Streptococcus*, or 0% *Lactobacillus*, 60.8% *Atopobium*, and 21.9% *Gardnerella*; these pregnancies were ongoing beyond 16 weeks at the time the article was published (Table 1) [107]. Consequently, the normal span of endometrial *Lactobacillus* levels should be reevaluated in fertile women when assessing its use as a biomarker for RIF.

Moreno et al. proposed that the primary factor affecting fertility may be the occurrence of pathogens in the uterine cavity rather than the necessity of a specific commensal taxon [72]. The absence of bacteria, including *Lactobacillus*, does not hinder implantation, further highlighting the role of pathogenic bacteria as a risk factor in reproduction. Indeed, the presence of pathogenic bacteria, such as *Actinobacteria*, *Atopobium*, *Bacillus halosaccharovorans*, *Bacillus simplex*, *Bifidobacterium*, *Burkholderia*, *Chryseobacterium*, *Corynebacterium coyleae*, *Delftia*, *Dialister*, *Dietzia*, *Enterococcus*, *E. coli*, *Gardnerella vaginalis*, *Glutamicibacter* spp., *Haemophilus*, *Hydrogenophaga*, *Klebsiella*, *Kocuria dechangensis*, *Leucobacter*, *Megasphaera*, *Microbacterium maritypicum*, *Micrococcus*, *Neisseria*, *Paenibacillus glucanolyticus*, *Prevotella*, *Pseudomonas*, *Psychrobacter*, *Ralstonia*, *Romboutsia*, *Roseiflexaceae*, *Schlegelella*, *Serratia marcescens*, *Sphingobacterium*, *Staphylococcus aureus*, and *Streptococci*, that would constitute part of a more diverse microbiota compared to their corresponding controls, was suggested to be related to reproductive failure (Table 1) [23,24,38,72,96,100,104,106,108,109,110,111,112,113,114,115]. Of all these pathogenic bacteria, *Actinobacteria*, *Fusobacteria*, *Streptococcus agalactiae*, *Klebsiella pneumoniae*, *Enterococcus faecalis*, *Neisseria gonorrhoeae*, *Gardnerella vaginalis*, and *Staphylococcus* are the major pathogens in the endometrium of patients with CE (Table 1) [72,74,116]. As it has been previously mentioned, this disease has been related to pregnancy failure since 14–41% of CE patients presented RIF, and 8–28% had RPL [74]. However, there is no consensus about which pathogenic bacteria(s) are undoubtedly correlated with infertility since the studies that report them do not completely agree [23,24,34,72,74,96,100,106,108,109,110,111,112,113,114,115]. Therefore, it has been postulated that the primary role of *Lactobacillus* spp. in reproduction is to prevent the establishment of pathogenic bacteria in the uterine cavity [72]. Further research is required to explore the mechanism through which pathogenic bacteria may influence embryo implantation. Also, it may be necessary to analyze microbiota at the species-level resolution to identify the true pathogenic bacteria of the endometrium since pathogenicity can vary between bacterial species; for example, *Streptococcus agalactiae* and *Streptococcus anginosus* belong to the same genus but may exhibit distinct behaviors in the endometrium [107]. Moreover, although *Lactobacillus* has been suggested as the dominant microbiome species that benefits and supports endometrial receptivity, the majority of the reports do not specify which *Lactobacillus* species may be most beneficial for pregnancy. However, *L. iners* has been described as offering a notable improvement in achieving favorable pregnancy outcomes [106].

In conclusion, a dysbiotic endometrial microbiome has been linked to implantation failure. This imbalance may trigger excessive immune stimulation, leading to inflammation and local tissue damage, which can result in transplantation failure, spontaneous preterm birth, and other adverse obstetric outcomes, such as growth restriction and stillbirth [115,117,118,119,120]. Nevertheless, the precise regulatory mechanisms remain unclear.

These data suggest that the EM may be regarded as an emerging factor contributing to implantation failure and/or pregnancy loss. As a result, microbial interventions such as antibiotics, probiotics, prebiotics, and microbial transplantation have been explored as strategies to modify the EM composition prior to a subsequent conception attempt in order to improve infertility treatment outcomes and IVF success [74]. Probiotics are living microorganisms that benefit the host by promoting a healthy balance of the microbiota, including preparations of *Lactobacillus* and *Bifidobacterium*. In contrast, prebiotics are nondigestible nutritive substances acting as substrates for protective endogenous bacteria to stimulate their growth and metabolism [66,121]. Lactoferrin (LF), an iron-binding glycoprotein found in human external secretions like breast milk, is one of the prebiotics described as being effective against numerous infectious diseases. LF exerts bacteriostatic effects due to its capacity to bind iron, making it unavailable to bacteria. LF effectively prevented preterm delivery in patients with a history of multiple miscarriages or early preterm delivery produced by refractory bacterial vaginitis by promoting the growth of *Lactobacilli* in their vaginal flora [122]. In the infertility clinical setting, antibiotic therapy, followed by a combination of prebiotics and/or probiotics, has been used to change the NLDM into LDM. Kadogami and cols. notified that in RIF patients, the combined use of a vaginal probiotic suppository (*L. gasseri*, *L. fermentum*, and *L. plantarum*) and vaginal antibiotics (metronidazole) transformed the NLDM into LDM (>90% *Lactobacillus* + *Bifidobacterium*) in 78.6% of the patients treated and that the therapeutic effects were unaffected by the prevailing bacteria present prior to the intervention [66]. Likewise, it has been reported in RIF patients that vaginal antibiotic (metronidazole) and vaginal probiotic formulations (*L. acidophilus La-14* and *L. rhamnosus HN001*) significantly enhanced *Lactobacillus* presence in the uterine microflora compared to oral formulations. Furthermore, it was observed that the *Lactobacillus* species that proliferated in the uterus after treatment was not limited to the dispensed species since *L. crispatus*, *L. jensenii*, *L. fermentum*, and *L. iners* were also detected. This indicates that the dispensed *Lactobacillus* species may have facilitated the creation of an intrauterine environment leading to *Lactobacillus* growth, allowing a subsequent proliferation of the originally existing *Lactobacillus* species in the uterine cavity [123]. Treatment with broad-spectrum antibiotics (amoxicillin or levofloxacin) in combination with LF and vaginal probiotics transformed the uterine NLDM into LDM. This change significantly increased pregnancy rates among LDM patients (61.3%) compared to the NLDM group (40%), with LDM defined as having ≥80% *Lactobacillus* spp. (Table 1) [124]. Similarly, other work reported that reproductive results in the immediate subsequent vitrified–warmed blastocyst transfer cycle were better in RIF women who resolved NLDM after LF supplementation compared to those whose local microbiota remained unchanged. Notably, the clinical pregnancy rate and the live birth rate for RIF women who increased the proportion of *Lactobacillus* species by at least 10% in EF samples were significantly higher (71.4% and 57.1%, respectively) than for RIF patients who did not overcome NLDM (22.2% and 11.1%, respectively) (Table 1) [121]. A similar increase in clinical pregnancy rate, ongoing pregnant rate, and live birth rate was observed in RIF patients who were treated with antibiotics (amoxicillin and clavulanic acid, or metronidazole) followed by vaginal probiotics to overcome NLDM, as compared to the non-treated RIF patients (Table 1) [125]. Additionally, Wei et al. informed that transvaginal *Lactobacillus* treatment significantly improved the clinical pregnancy rate in women with prior failed cycles and a low initial proportion of *Lactobacillus* (Table 1) [104].

**Table 1:** Microbiota composition in endometrium of patients undergoing assisted reproductive technology (ART), and positive or negative outcomes of the treatment.

Ref.	Patient Profile and Treatment Approach for Correcting the NLDM (the Latter, When Indicated)	Detected Microbiota Composition	Positive Outcomes (Ongoing Pregnancy Included)	Negative Outcomes (Non-Pregnant or Decreased Clinical Pregnancy Rate)
[26]	35 infertile subjects undergoing IVF 35 fertile women at pre-receptive (LH+2) and receptive phases (LH+7) LDM (>90% *Lactobacillus* spp.) NLDM (<90% *Lactobacillus* spp. with >10% other bacteria)	166 different OTUs *Lactobacillus* 71.7%, *Gardnerella* 12.6%, *Bifidobacterium* 3.7%, *Streptococcus* 3.2%, *Prevotella* 0.866%. Others: *Bacillus*, *Bacteroides*, *Bifidobacterium*, *Blautia*, *Clostridiales*, *Clostridium*, *Escherichia*, *Faecalibacterium*, *Gardnerella*, *Lachnospiraceae*, *Propionibacterium*, *Pseudomonas*, *Roseburia*, *Ruminococcus*, *Veillonella.* EM was not hormonally regulated during the acquisition of endometrial receptivity.	LDM was associated with significant increases in implantation rate: 60.7% *; pregnancy rate: 70.6% *; ongoing pregnancy rate: 58.8% *; and live birth rate: 58.8% *.	NLDM was linked to significant reductions in implantation rate: 23.1%; pregnancy rate: 33.3%; ongoing pregnancy rate: 13.3%; and live birth rate: 6.7%.
[60]	33 IVF patients 26 (79%) Caucasian 5 (15%) Asian 1 (3%) African American 1 (3%) Hispanic	278 different genera *Flavobacterium* and *Lactobacillus* constitute the predominant bacterial genera observed in both groups; Other detected bacterium were *Acidovorax*, *Acinetobacter*, *Bdellovibrio*, *Blvii28*, *Candidatus aquiluna*, *Cellvibrio*, *Chryseobacterium*, *Clostridium*, *Curvibacter*, *Delftia*, *Fluviicola*, *Janthinobacterium*, *Limnohabitans*, *Methylotenera*, *Microbacterium*, *Paucibacter*, *Paudibacter*, *Pedobacter*, *Polosinus*, *Polynucleobacter*, *Pseudomonas*, *Salinibacterium*, *Shuttleworthia*, *Spirocheta*, *Streptococcus*, *Sulfospirilum*, *Sulfuricurvum*	18 patients had ongoing pregnancies.	15 non-pregnant patients. Certain major species seemed to differ based on the outcome, although these differences were not statistically significant.
[62]	40 reproductive-aged Chinese women, 10 infertile patients. Participants with an IUD, vaginal inflammation, acute inflammation, suspected cervical or endometrial neoplasia, or endocrine or autoimmune disorders were excluded. Additionally, participants had no recent use of hormones, antibiotics, or vaginal medications; no cervical treatment, endometrial biopsy, IUD removal, or hysteroscopy within the past week; no douching within 5 days; and no sexual intercourse within 48 h. None of the participants were pregnant, lactating, or menstruating at the time of sampling.	Genera with the highest abundance: *Bacteroides*, *Elizabethkingia*, *Lactobacillus*, *Methylotenera*, *Porphyrobacter*, *Prevotella*, *Pseudochrobactrum*, *Rheinheimera*, *Streptophyta.* Differences between endometrial and vaginal microbiota The uterine cavity microbiota may help to distinguish infertile patients from healthy individuals and could play a role in infertility.		*L. iners* and *L. crispatus* showed a significant reduction in the uterine cavity of 10 infertile patients.
[72]	A total of 342 infertile patients undergoing ART were enrolled across 13 centers on three continents, with the following demographics: Caucasian (57.3%), East Asian (14.0%), Hispanic (11.4%), and other ethnicities (17.3%). Personalized assessment of window of implantation (and optimal time frame for embryo transfer) by ERA test. No antibiotics in the last 3 months before sample collection, no uterine pathologies, no women with serious or uncontrolled bacterial, fungal, or viral infections were included.	Identified microbiota composition in EF partially reflected that in endometrial biopsy. But association with clinical outcome was consistent.	*Lactobacillus* was consistently enriched in patients who achieved live birth. Some commensal bacteria, including *Cupriavidus*, *Finegoldia*, *Microbacterium*, and *Tepidimonas* were positively correlated with live birth outcomes.	Reduced levels of *Lactobacillus* spp. accompanied by an increased presence of *Anaerococcus*, *Atopobium*, *Bifidobacterium*, *Chryseobacterium*, *Escherichia*, *Bacillus*, *Gardnerella*, *Haemophilus*, *Klebsiella*, *Neisseria*, *Propionibacterium*, *Staphylococcus*, and *Streptococcus* were linked to either no pregnancy or clinical miscarriage.
[74]	94 IVF Asian patients 25 patients with chronic endometritis (CE) 69 patients with non-chronic endometritis (NCE)	Ten most abundant phyla: *Acidobacteria*, *Actinobacteria*, *Bacteroidetes*, *Chloroflexi*, *Deinococcus-Thermus*, *Firmicutes*, *Fusobacteria*, *Gemmatimonadetes*, *Patescibacteria*, *Proteobacteria.* Ten most abundant genera: *Chelativorans*, *Gardnerella*, *Halomonas*, *Lactobacillus*, *Lysobacter*, *Mitochondria*, *Pelagibacterium*, *Pseudomonas*, *Sneathia*, *Sphingomonas.*	CE group with clinical pregnancy *n* = 8 (32%) NCE group with clinical pregnancy *n* = 41 (59.4%) * Relative abundance of *Proteobacteria* * and Acidobacteria * significantly higher in pregnant NCE group vs. non-pregnant CE group.	CE group with pregnancy failure *n* = 17 (68%) NCE group with pregnancy failure *n* = 28 (40.6%) * Relative abundance of *Actinobacteria* * significantly higher in non-pregnant CE group vs. pregnant and non-pregnant NCE groups. Relative abundance of *Fusobacteria* * significantly higher in pregnant CE group vs. pregnant and non-pregnant NCE groups. Relative abundance of *Gardnerella* * significantly increased in CE group vs. NCE and was elevated in non-pregnant groups vs. pregnant group in both CE and NCE groups.
[96]	45 Caucasian females who underwent ART: 27 women RIF and 18 women without RIF (control).		Most frequent genera in control patients: *Anaerobacillus* (0.22%), *Bacillus* (0.02%), *Bifidobacterium* (0.11%), *Burkholderia* (0.11%), *Citrobacter* (0.01%), *Delftia* (0.05%), *Dialister* (0.06%), *Gardnerella* (1.07%), *Lactobacillus* (97.96%), *Lysinibacillus* (0.03%), *Prevotella* (0.00%), *Ralstonia* (0.45%), *Streptococcus* (0.05%).	Most frequent genera in RIF patients: *Anaerobacillus* (0.63%), *Bacillus* (0.03%), *Bifidobacterium* (0.00%) *, *Burkholderia* (0.45%), *Citrobacter* (0.07%), *Delftia* (0.23%), *Dialister* (0.15%) *, *Gardnerella* (2.18%), *Lactobacillus* (92.27%) *, *Lysinibacillus* (0.03%), *Prevotella* (2.19%) *, *Ralstonia* (1.16%), *Streptococcus* (0.18%) *.
[102]	102 Japanese infertile patients (79 IVF and 23 non-IVF) 7 healthy volunteers	Major taxonomies present in samples: *Aerococcus*, *Atopobium*, *Bifidobacterium*, *Enterococcus*, *Escherichia*, *Gardnerella*, *Lactobacillus*, *Prevotella*, *Sneathia*, *Staphylococcus*, *Streptococcus*, *Ureaplasma.* Endometrial microbiome of the healthy women exhibited high stability both between and within cycles. Percentage of patients with LDM (>90% *Lactobacillus* spp.): IVF 38% (30/79) * Non-IVF 73.9% (17/23) Healthy 85.7% (6/7) Median percentage of the endometrial *Lactobacilli* in patients: IVF 63.90 ± 41.43% of *Lactobacilli* * Non-IVF 96.20 ± 34.61% of *Lactobacilli* Healthy 99.50 ± 15.85% of *Lactobacilli*	18 patients pregnant: 3 natural conception, 15 FBT. Median percentage of the endometrial *Lactobacilli* in pregnant individuals: 96.45% ± 33.61%. LDM endometrium might favor implantation. 7 NLDM cases pregnant (6 IVF, 1 non-IVF): 5 cases are ongoing, 1 early miscarriage, 1 was lost to follow-up.	
[104]	60 Chinese patients with previous failed cycles: control group (*n* = 30) and treatment group (*n* = 30). A live *Lactobacillus* was given intravaginally for 30 consecutive days prior to the initiation of the FET cycle.	A small percentage of *Lactobacillus* (2.7%). Three most dominant microbiota: *Rhodococcus* (23.7%), *Pseudomonas* (4.9%), and *Achromobacter* (4.1%) No significant modifications in the EM conformation were recorded among the clinical pregnant, miscarriage, and non-pregnant groups.	Clinical pregnancy rate: treated 66.7% (20/30) * Transvaginal *Lactobacillus* supplementation significantly increased the clinical pregnancy rate. Associated with clinical pregnancy: *Achromobacter*	Clinical pregnancy rate: Control 36.7% (11/30): The miscarriage frequency presented no variance amongst the two groups. Associated with miscarriage: *Corynebacterium*, *Enterobacter*, *Nocardioides*, *Roseifexaceae.* Negative correlations with clinical pregnancy: *Chryseobacterium*, *Psychrobacter*, *Romboutsia*, *Roseifexaceae*.
[101]	48 women undergoing IVF with FET with no antibiotic treatment in the 3 months preceding the fertility treatment.	Clear dominance of *Lactobacillus* genus in the endometrial microbiome.	21 women pregnant, 5 (23.81%) women with RIF. Greater abundance of *Anaerobacillus* spp., *Burkholderia* spp., *Gardnerella* spp., *Lactobacillus* spp., although the difference was not significant. Greater abundance of *L. iners*, *L. jensenii*, and *Ralstonia* spp. in women without RIF. Women with NLDM, characterized by a relative abundance of over 80% *Lactobacillus* spp. in the endometrium, presented favorable pregnancy outcomes.	27 women not pregnant, 18 (66.66%) women with RIF. Greater abundance of *Delftia* spp., *Prevotella* spp., *Ralstonia* spp., and *Streptococcus* spp., although the difference was not significant. Greater abundance of *Prevotella* spp., *L. helveticus* and *Sneathia amnii* in women with RIF.
[100]	93 infertile women IVF. Exclusion criteria included pelvic inflammatory disease, fibroids, endometrial polyps or septate uterus, endometrial hyperplasia or cancer, failure in oocyte recovery, poor quality blastocytes, cervicovaginal infections, sexually transmitted disease, and antimicrobial treatment in the last 4 weeks.	Microbiota phyla detected (species)*:* 87.76% *Firmicutes* (*Bacillus halosaccharovans*, *Bacillus simplex*, *Enterococcus faecalis*, *Escherichia coli*, *L. crispatus*, *L. fermentum*, *L. gasseri*, *L. iners*, *L. jensenii*, *L. jonsonii*, *L. paracasei*, *L. rhamnosus*, *Paenibacillus glucanolyticus*, *Paenibacillus* spp., *S. aureus*, *S. capitis*, *S.epidermidis*, *S. hominis*, *S. pasteuri*, *S. warneri; Se. agalactiae*, *Se. anginosus*, *Se. mitis*, *Se. oralis*, *Se. salivarius*, *Se. urinalis*, *Se. vestibularis*). 27.94% *Proteobacteria* (*Alcaligenes faecalis*, *Citrobacter koseri*, *Enterobacter kobei*, *Haemophilus haemolyticus*, *Klebsiella pneumoniae*, *Neisseria subflava*). 10.29% *Actinobacteria* (*Bifidobacterium scardovii*, *Corynebacterium coyleae*, *Corynebacterium* spp., *Gardnerella vaginalis*, *Microbacterium maritypicum*) 8.82% *Ascomycota* (*C. albicans*, *C. glabrata*, *C. krusei*, *C. lusitaniae*, *C. parapsilosis*)	35 patients (37.6%) achieved clinical pregnancy. 27 patients (29%) presented endometrial bacterial colonization and 8 (8.6%) showed no microbial development. No differences in reproductive outcome between these two group of patients. Positive impact of *Lactobacillus* spp. on ongoing pregnancy rate.	58 patients (62.4%) non-pregnant. 41 patients (44.1%) presented endometrial bacterial colonization and 17 (18.3%) showed no microbial development. Higher proportion of the following families in non-pregnant patients: *Enterobacteriaceae* and *Staphylococcaceae.* The Phylum *Actinobacteria* was exclusively found in non-pregnant patients. Specific species found in non-pregnant patients: *Bacillus halosaccharovorans*, *Bacillus simplex*, *Bifidobacteria scardovii*, *Corynebacterium coyleae*, *Gardnerella vaginalis*, *Haemophilus haemolyticus*, *Microbacterium maritypicum*, *Paenibacillus glucanolyticus.*
[105]	185 infertile Japanese patients. EMMA and ALICE evaluated 40 patients pattern 1 (*Lactobacillus* > 90%), 8 patients pattern 2 (*Lactobacillus* < 90% and negative for bacterial pathogens producing CE), 32 patients pattern 3 (*Lactobacillus* < 90% and positive for bacterial pathogens producing CE), 49 patients pattern 4 (minor dysbiotic microbiome profile), 56 patients pattern 5 (ultralow biomass microbiome).	Pathogens causing CE included: *Chlamydia*, *Enterococcus*, *Escherichia* *Klebsiella*, *Mycoplasma*, *Staphylococcus*, *Streptococcus*, *Ureaplasma.*	111 patients receptive ERA group. Normal microbiome (pattern **1**) was significantly associated with receptive endometrium.	74 patients pre-receptive ERA group. Aging and ultralow biomass EM (pattern **5**) were both significantly linked to a pre-receptive endometrium.
[106]	34 Caucasian women personalized hormonal stimulation, IVF. Infertility was attributed to tubal occlusion (7/34), endometriosis (3/34), ovulatory disorder (9/34), or idiopathic infertility (13/34) persisting for at least 1 year.	319 bacterial species identified: *Actinobacteria*, *Bacteroidetes*, *Cyanobacteria*, *FBP*, *Firmicutes*, *Proteobacteria*, *Thermi*, *Verrucomicrobia.*	4/34 pregnant. Predominant presence of *Lachnospiraceae* and *Enterobacteriaceae* with a significant reduction in bacterial richness.	30/34 non-pregnant. *Lactobacilli* were identified only in the group with failed in vitro fertilization outcomes. *Kocuria dechangensis* was the only endometrial species with a significantly increased relative proportion in non-pregnant women.
[110]	70 IVF patients: 43 (61%) Caucasian, 12 (17%) Asian, 1 (1.4%) African American, 4 (5.6%) Hispanic, 11 (15%) unknown.	50 different genera: *Achromobacter*, *Acinetobacter*, *Actinomyces*, *Aerococcus*, *Alloscardovia*, *Anaerococcus*, *Bacillus*, *Bdelovrio*, *Bifidobacterium*, *Bosea*, *Brevundimonas*, *Brochothrix*, *Burkholderia*, *Caloramator*, *Clostridium*, *Comamonas*, *Corynebacterium*, *Enterococcus*, *Escherichia*, *Facklamia*, *Finegoldia*, *Fusobacterium*, *Gardnerella*, *Herbaspirilum*, *Hydrogenophylus*, *Jonquetella*, *Kouleothrix*, *Lactobacillus*, *Lysinbacilus*, *Methylobacterium*, *Moraxella*, *Moritella*, *Moryella*, *Paenibacillus*, *Peptoniphilus*, *Petrobacter*, *Photobacterium*, *Prevotella*, *Pseudomonas*, *Raistonia*, *Serratia*, *Sphingomonas*, *Staphylococcus*, *Stenotrophomonas*, *Streptococcus*, *Thermicanus*, *Varibacterium*, *Veillonella*, *Vogesella* 33 patients > 90% *Lactobacillus* abundance 50 patients > 70% *Lactobacillus* abundance	Not evaluated	Not evaluated
[111]	All Japanese population: 28 infertile patients with RIF history and 18 infertile patients undertaking their first IVF cycle (control group). CE was detected in 6 (21.4%) RIF patients and in 2 (11.1%) control.	26,725 OTUs *Aerococcus*, *Atopobium*, *Bacillus*, *Bifidobacterium*, *Burkholderia*, *Corynebacterium*, *Dialister*, *Enhydrobacter*, *Enterococcus*, *Exiguobacterium*, *Finegoldia*, *Fusobacterium*, *Gardnerella*, *Lactobacillus*, *Leucobacter*, *Megasphaera*, *Mobiluncus*, *Mycoplasma*, *Nesterenkonia*, *Peptoniphilus*, *Prevotella*, *Pseudoalteromonas*, *Shewanella*, *Sneathia*, *Staphylococcus*, *Streptococcus*, *Ureaplasma*, *Variovorax*, *Vibrio.* EF microbiota significantly differed between the RIF and the control group (*p* = 0.0089). Percentage of patients with LDM (>90% *Lactobacillus* spp.): RIF 64.3% (18/28) Control 38.9% (7/18)	*Burkholderia* was absent from all EF microbiota samples in the control group.	*Burkholderia* was detected in 7 of 28 (25%) * RIF patients.
[112]	145 patients with RIF 21 controls RIF patients without endometrial polyps, submucosal myomas, intrauterine adhesions, thrombophilia, endocrinologic abnormalities, collagen disease, recent antibiotic treatment or parental chromosomal imbalances, or translocations.	131 bacterial species detected in endometrial samples. Relative quantity of endometrial *Lactobacillus* did not change significantly between the RIF and control groups (51.2 ± 37.5% and 51.6 ± 38.3%, respectively).	Bacterial abundance: *Atopobium* (0.1 ± 0.2), *Burkholderia* (0.1 ± 0.2), *Delftia* (0 ± 0.1), *Dietzia* (0 ± 0), *Enterococcus* (0 ± 0), *Gardnerella* (0.6 ± 1.6), *Hydrogenophaga* (0 ± 0), *Leucobacter* (0.1 ± 0.2), *Megasphaera* (0 ± 0), *Micrococcus* (0 ± 0), *Prevotella* (0 ± 0.1), *Ralstonia* (0 ± 0.1), *Schlegelella* (0 ± 0), *Sphingobacterium* (0 ± 0)	Bacterial abundance: *Atopobium* (2.1 ± 9.4) *, *Burkholderia* (0.5 ± 1.3) *, *Delftia* (0.2 ± 0.3) *, *Dietzia* (0.1 ± 0.5) *, *Enterococcus* (0.1 ± 0.3) *, *Gardnerella* (5.3 ± 16.3) *, *Hydrogenophaga* (0.1 ± 0.3) *, *Leucobacter* (0.2 ± 0.6) *, *Megasphaera* (0.8 ± 3.2) *, *Micrococcus* (0.1 ± 0.7) *, *Prevotella* (0.7 ± 2.6) *, *Ralstonia* (0.3 ± 1.2) *, *Schlegelella* (0.4 ± 1.1) *, *Sphingobacterium* (0.3 ± 1.1) *.
[113]	130 infertile patients. Group I: 39 women with the first IVF attempt with ovarian stimulation. Group II: 27 RIF patients with ovarian stimulation and embryo transfer. Group III: 64 RIF patients with frozen-thawed embryo transfer in natural cycle.	*Lactobacilli* (14 species) were dominant across all groups, with *L. crispatus*, *L. jensenii*, *L. vaginalis* being the most prevalent.	The pregnancy rate per embryo transfer was 51.3% in group I, higher than 29.6% in group II, and 35.9% in group III, although the differences were not statistically significant. Group I showed a significantly higher isolation frequency of obligate anaerobic microorganisms and *G. vaginalis* than group III.	*Enterobacteria* and *Staphylococci* were more frequently observed in patients from group III compared to those in groups I and II. *Streptococci* were more commonly detected in patients from groups II and III than in those from group I.
[114]	177 Caucasian infertile patients. Participants had not used hormonal contraceptives, antibiotics, or probiotic, or prebiotic, or synbiotic formulations for at least 3 months prior to the examination. No malformations of the uterus and fallopian tubes, no endometriosis, no vaginal infections.	105 strains of bacteria. 10 bacteria most common in patients: *Bifidobacterium longum*, *Escherichia coli*, *Gardnerella vaginalis*, *L. gasseri*, *L. helveticus*, *L. iners*, *L. jensenii*, *L. paracasei*, *L. reuteri*, *Staphylococcus aureus.*	67 women were pregnant based on β-hCG levels 14 days after embryo implantation. In 65 patients (97%), the pregnancy ended in childbirth, while the remaining two suffered a miscarriage.	*E. coli* and *Gardnerella vaginalis* reduced the protective effect of *Lactobacilli* before, during, and after embryo implantation.
[115]	30 infertile patients undergoing IVF. No recent history of inflammatory disease, chronic endometritis, antibiotic treatment, moderate to severe endometriosis, adenomyosis, uterine hyperplasia, or endometrial polyps.	2168 OTUs were identified. *Lactobacillus* genus was not significantly different between pregnant and non-pregnant groups.	Pregnant women (*n* = 16). 39 (14.39%) unique species found. *Bosea* spp. was detected frequently in more than 30% of the samples. More frequent genera: *Ralstonia genus* (28.89%), *Lactobacillus* spp. (14.44%), *Pseudomonas* spp. (0.77%), *Delftia* spp. (0.21%).	Non-pregnant women (*n* = 14). 62 (22.88%) unique species found. Bacteria that occur frequently: *Acetomicrobium* spp., *Bacteroides* spp., *Cutibacterium granulosum*, *Isoptericola* spp., *Marivivens* spp., *Syntrophomonas* spp. More frequent genera: *Ralstonia genus* (33.88%), *Lactobacillus* spp. (10.16%), *Ureaplasma* spp (1.27%), *Faecalibacterium* spp. (0.89%), *Pseudomonas* spp. (0.87%), *Delftia* spp. (0.72%). Significantly enriched *Delftia* spp., *Glutamicibacter* spp., *Serratia marcescens*, *Staphyloccocus* spp.
[116]	80 asymptomatic Chinese women with RIF: 40 patients non-CE and 40 patients CE. CE patients were treated with doxycycline (100 mg twice daily for 14 days). After treatment, the 40 CE patients were CD138-negative by immunohistochemistry.	*Lactobacillus* is non-predominant genera of EM Top microbiota phylum: *Acinetobacter*, *Lactobacillus*, *Pseudomonas*, *Rhodococcus.* Associated with CE: *Aminicenantales*, *Chloroflexaceae*, *Proteobacteria.* Associated with non-CE *Acinetobacter*, *Herbaspirillum*, *Lactobacillus*, *Micrococcaceae*, *Ralstonia*, *Shewanela.*	Clinical pregnancy rate Non-CE group 62.5% (25/40) * Associated with clinical pregnancy: *Achromobacter*, *Acinetobacter*, *Lactobacillus*, *Proteobacteria.*	Clinical pregnancy rate CE group 37.5% (15/40) There was no variation in the miscarriage rate between the two groups. Correlated with miscarriage: *Enterococus*, *Gardnerella*, *Phyllobacterium*, *Pseudomonas.* Correlated with non-pregnancy: *Clostridium*, *Prevotella*, *Romboutsia*, *Streptococcus.*
[121]	117 RIF women and 55 infertile women without RIF LDM (>90% *Lactobacillus*-dominant microbiota) and NLDM (≤90% *Lactobacillus* microbiota). Patients with NLDM EF treated with oral lactoferrin supplementation (700 mg/day for a minimum of 28 consecutive days). Improved EF microbiotas are patients who increased 10% or more the proportion of *Lactobacillus* species in EF samples after lactoferrin treatment.	No identification of single microorganisms or characterization of the local microbiota associated with NLDM.	RIF group with improved EF microbiota: Clinical pregnancy rate: 71.4%(10/14) * Live birth rate: 57.1% (8/14) *	RIF group with unimproved EF microbiota: Clinical pregnancy rate: 22.2% (2/9) Live birth rate: 11.1% (1/9)
[124]	92 Asian IVF patients (90 Japanese, 1 Korean, and 1 Chinese). Nine NLDM patients treated with amoxicillin or levofloxacin, followed by combination of prebiotics (lactoferrin) and/or probiotics.	Major taxonomies present in samples: *Aerococcus*, *Atopobium*, *Bifidobacterium*, *Enterococcus*, *Escherichia*, *Gardnerella*, *Lactobacillus*, *Prevotella*, *Sneathia*, *Staphylococcus*, *Streptococcus*, *Ureaplasma.* 62 patients with LDM (≥80% *Lactobacillus* spp.) 30 patients with NLDM (< 80% *Lactobacillus* spp.)	Pregnancy rate per patient: LDM 38 patients (61.3%) * NLDM 12 patients (40%) All nine NLDM patients became LDM, and five patients achieved pregnancies (three ongoing and two miscarriages). LDM endometrium might benefit implantation	Non-pregnant patients: LDM 32 patients (47.1%) NLDM 14 patients (45.2%) In these patients, the median percentage of *Lactobacilli* was 14.75% (range 0–78.6%), *Gardnerella* (11.0–98.8%), *Atopobium* (3.8–97.3%), *Streptococcus* (65.4–81.5%).
[125]	195 Japanese RIF patients: 131 EMMA evaluated (initially, 67 patients LDM, 64 patients NLDM) and 64 not evaluated (control group). Patients were excluded if they had intrauterine lesions, untreated hydrosalpinx, an allergy to antibiotics or inability to adhere to antibiotic treatment, or received antibiotic treatments within 3 months prior to sample collection. Antibiotics were chosen based on the pathogens identified in the EMMA test and the clinical profile of each patient. Metronidazole (500 mg twice a day for 7 days) when *Gardnerella* was detected. Amoxicillin and clavulanic acid (500–125 mg every 8 h for 8 days) when *Streptococcus* > 10% of EM. Vaginal suppositories containing *Lactobacillus* strains administered after antibiotic treatment for 7–10 days or for 10–17 days from day 5 of their FET cycle. All control group patients were given intravaginal probiotic treatment for 7–10 days, beginning on the 5th day of their FET cycle. Al 64 NLDM patients treated achieved LDM. Median percentage *Lactobacillus* spp: before treatment 25.8%, after treatment 90.8%.	*Lactobacillus* spp. detected in all patients. More frequently detected genera: *Atopobium*, *Bifidobacterium*, *Gardnerella*, *Streptococcus.*	Patients with LDM Clinical pregnancy rate: 64.5% (79/131) * Ongoing pregnant rate: 48.9% (64/131) * Live birth rate: 48.9% (64/131) * Weeks of gestation for single births: 38.8 ± 1.71 *	Patients not evaluated with EMMA (control group): Clinical pregnancy rate: 33.3% (25/64); Ongoing pregnant rate: 32.8% (21/64); Live birth rate: 31.2% (20/64); and Weeks of gestation for single births: 37.6 ± 3.42.

ALICE, analysis of infectious chronic endometritis; ART, assisted reproductive technology; βhCG: β-human chorionic gonadotropin; *C*, *Candida*; CE, chronic endometritis; EM, endometrial microbiota; EMMA, endometrial microbiome metagenomics analysis; ERA, endometrial receptivity analysis; EF, endometrial fluid; ET, embryo transfer; FET, frozen embryo transfer; IVF, in vitro fertilization; IUD, intrauterine device; *L*, *Lactobacillus*; LDM, *Lactobacillus*-dominated microbiota; LH+2, pre-receptive phase, two days after the luteinizing hormone surge; LH+7 receptive phase, seven days after the luteinizing hormone surge; NLDM, non-*Lactobacillus*-dominated microbiome; OTU, operational taxonomic units; RIF, recurrent implantation failure; RPL, repeated pregnancy loss; *S*, *Staphylococcus*; and *Se*, *Streptococcus*. * *p* < 0.05 between groups.

In conclusion, maintaining an adequate EM is essential for improving pregnancy results in RIF patients. Personalized treatment strategies guided by microbial 16S rRNA gene sequencing can help establish an optimal intrauterine environment and enhance IVF success rates in RIF cases. Additionally, the use of microbial 16S rRNA gene sequencing can minimize the need for broad-spectrum antibiotics, thereby reducing the physical, psychological and economic loads on patients. However, further studies are necessary to analyze the mechanisms by which pathogenic bacteria affect embryo implantation.

## 6. Effect of Bisphenols on Reproduction

Human exposure to BPA and its analogs is a public health concern because bisphenols have the ability to bind to membrane and nuclear receptors such as androgen, estrogen, and thyroid receptors, causing endocrine disruption, tumors, adverse reproductive outcomes, and transgenerational effects [12]. BPA has been associated with alterations in hormonal levels, with elevated levels of testosterone (T), and E_2_ and P_4_ detected in the urine of adolescent women with impaired reproductive functions or with polycystic ovary syndrome (PCOS) [126,127]. Studies demonstrating impaired ovarian and uterine function indicated that elevated urinary BPA levels are linked to a higher possibility of developing PCOS and a decreased antral follicle count [127,128]. Additionally, urinary BPA levels were negatively correlated with both the number of oocytes recovered in women undertaking IVF and the serum E_2_ levels [129]. Also, an association between high urinary BPA concentration and increased serum T, E_2_, and pregnenolone levels was reported in girls diagnosed with precocious puberty [130]. Moreover, increased urine BPA concentrations were associated with reduced fecundity in Chinese women attempting to conceive [131]. Other studies have demonstrated that elevated serum and urinary BPA levels are linked to a higher risk of miscarriage [132,133]. Furthermore, high BPA levels in maternal blood, urine, or amniotic fluid have been related to reduced weight gain throughout pregnancy and low birth weight [134]. Likewise, BPA was consistently linked to preeclampsia in several studies [135]. Regarding uterine morphology, BPA has been associated with non-ovarian pelvic endometriosis, as an increment in urinary concentrations of the endocrine disruptor was found in these patients [136,137]. Taken together, these results suggest a link between BPA exposure and impaired reproductive function in women.

BPA’s effects on reproduction and, specifically, on uterine physiology have been the focus of numerous studies [137]. Research in rodents has shown that exposure to BPA throughout the critical phase of blastocyst implantation interferes with pregnancy. This may be linked to disturbance in various markers of uterine implantation, particularly those regulated by ovarian steroid hormones, leading to fewer implantation sites and lower pregnancy rates. These effects could result from a discrepancy between the timing of blastocyst formation and the uterine receptivity window or from the direct interference with uterine receptivity due to the estrogenic properties of BPA [137,138,139,140]. The expression of blastocyst implantation markers like HOXA10, Mucin 1, E-cadherin, and TJ proteins (occludin) have been reported to be altered by BPA [140,141]. Moreover, BPA exposure has been demonstrated to impact ovarian function. Prenatal exposure to BPA prevented germ cell nest breakdown in the ovaries of F1 generation in mice, reduced the number of primordial, primary, preantral, and total healthy follicle at post-natal day 21, and reduced E_2_ levels in female rats exposed for 1 year, indicating that BPA directly targets the ovaries [142,143]. Furthermore, studies have shown that BPA disturbs the hypothalamic–pituitary–gonadal (HPG) axis in mice, rats, and zebrafish [144,145,146].

Recent studies have concentrated on the effects of compounds structurally similar to BPA on endocrine disruption [147]. For instance, exposure to bisphenol analogs negatively impacts ovarian steroidogenesis. In zebrafish, BPS, BPF, BPB, and BPAF have been shown to disrupt the normal function of the HPG axis, leading to an aberrant production of the luteinizing hormone (LH) and the follicle-stimulating hormone (FSH) [148]. In rodents, exposure to BPS, BPF, and BPB adversely affected the secretion of hormones such as E_2_, P_4_, and T [149]. Additionally, BPE, BPS, BPF, and BPAF negatively impact the transcription of several genes critical for ovarian steroidogenesis, including StAR, Cyp17a1, 3β-HSD, and Cyp19a1 [150]. Prenatal or prepubertal exposure to BPB, BPF, and BPS may reduce the number of antral follicles by activating apoptosis and autophagy pathways, ultimately lowering the production of E_2_ and P_4_, and increasing the number of atretic and cystic follicles [149]. Bisphenol analogs can mimic E_2_ by interacting with the estrogen receptor (ER) to form complexes that bind to the nuclear DNA response element. This binding activates downstream transcription factors, initiating estrogenic effects. BPA, BPF, and BPS behave as partial agonists for human ERα (hERα) and full agonists for hERβ, with BPA being the most potent, followed by BPF and then BPS for both receptors [151]. Regarding androgenic activity, BPA and BPF function as full androgen receptor antagonists, with BPA showing stronger affinity [151]. Both BPA and BPS act as weak androgen receptor agonists [151]. In rats, exposure to BPS and BPF stimulated uterine growth, demonstrating estrogenic activity [152,153]. Additionally, neonatal exposure to BPS has been associated with delayed puberty onset, disrupted estrous cycles, increased body weight, and significantly reduced absolute and relative uterine weights. A decrease in plasma P_4_, LH and FSH concentrations was also observed, while T and E_2_ plasma concentrations were significantly increased. An increase in the number of cystic and atretic follicles in the ovaries was also reported [154]. In mice, a PCOS-like condition after exposure to BPS has been reported [155]. PCOS has been known to cause infertility in humans. BPS and BPE triggered follicular development problems in mice. Along the same line, a significant decline in pregnancy rates was observed, along with a reduced number of live births, an increase in deceased pups, and increased complications during childbirth [150]. Additionally, studies have reported that BPS and BPF can impair the receptivity of human endometrial epithelial cell in vitro by modulating steroid hormone receptor function, ultimately disrupting embryo implantation. These EDs suppressed spheroid (blastocyst surrogate) attachment to human endometrial epithelial cells through the regulation of genes that control endometrial receptivity, such as progesterone receptor (PR), olfactomedin 1, and thrombospondin 1 [156]. There are also several epidemiological studies suggesting that BPA alternatives (BPS or the mixture of BPS, BPF, and BPA) can alter the duration of pregnancy leading to preterm births [157,158]. In addition, in rat models, more than 80% of pregnant dams exposed to BPF during gestation experienced spontaneous abortions [159].

Collectively, these studies indicate that bisphenols detrimentally affect the reproductive tract and fertility of animals and humans. However, there are no reports that investigate the effects of bisphenols on the uterine microbiota, but some inferences can be gleaned by looking at the gut.

## 7. Bisphenols and the Gut Microbiota

The gut microbiota represents the largest microbial community of the human body. As a vital “microbial organ”, gut microbiota plays a role in host health, in nutrient digestion and absorption, growth, development, and disease prevention [160]. In addition, host phenotypic status and/or resident microbiota may influence the pharmacokinetics of EDs, including uptake, absorption, distribution, and metabolism [161]. In humans, the gut microbiota is an abundant ecosystem of highly diverse microorganisms where *Firmicutes* and *Bacteroidetes* are the dominant phyla (representing at least 90%), followed by *Proteobacteria* and *Spirochaetae* [162]. In rodents, the five dominant phyla—*Verrucomicrobia*, *Bacteroidetes*, *Firmicutes*, *Proteobacteria*, and *Actinobacteria*—collectively represent at least 99% of all detected phyla [163]. The *Bacteroidetes* phylum primarily generates acetate and propionate, whereas the *Firmicutes* phylum is the main producer of butyrate.

Studies in rodents have shown evidence that BPA exposure (either by direct ingestion or through the pregnant mother to the offspring) affects the gut microbiota by decreasing the diversity [13,164,165,166,167]. In these animals, the gut microbiota was similar to the one found in animals fed a high-fat diet, presenting a significant increase in microbial dysbiosis indicators, such as *Proteobacteria* (mainly *Epsilonproteobacteria*, *Parasutterella*, and *Helicobacteriaceae* (*Helicobacter ganmani*)) (Table 2) [13,164]. Also, in BPA-exposed rodents, a significant decrease in *Actinobacteria (Bifidobacterium* spp.), *Bacteroidetes (Prevotellaceae NK3B31 group*, *Rikenellaceae RC9 gut group*, *Prevotella 1*, *Prevotella 2*, *Prevotella 9*, *Parabacteroides*, *Muribaculum*, and *Alloprevotella*), *Firmicutes (Lactobacillus intestinalis*, *Tenericutes*, *Allobaculum*, *Marvinbryantia*, *Christensenellaceae_R_7_group*, *Streptococcus*, and *Clostridia* (*Clostridium viridae*, *Eubacterium dolichum*, *Coprococcus comes*, *Ruminococcaceae NK4A214 group*, *Ruminococcaceae UCG 002*, *Ruminococcaceae UCG 010*, *Eubacterium coprostanoligenes group*, *Oscillospira*, *Clostridium butyricum*, and *Clostridium Cluster XIVa*)), and *Verrucomicrobia* (*Akkermancia*), which are indicators of a healthy intestinal barrier function, has been reported, as compared to the control animals [13,166,167,168,169,170,171,172,173] (Table 2).

In contrast, experiments with other rodents have shown no changes in gut microbiota diversity and even increases in proportions of *Firmicutes* (*Lactobacillaceae*, *Lactobacillus* spp., *L. reuteri*, *Subdoligranulum*, *Blautia*, *Veillonella*, *Bacillales*, *Faecalibaculum*, and *Clostridia* (*Ruminococcaceae*, *Oscillospira* spp., *Oscillibacter*, *Ruminococcaceae NK4A214 group*, *Ruminococcaceae UGG 009*, *Ruminococcaceae UCG-010*, *Clostridium perfringens*, and *Clostridium ruminantiums*)), *Verrucomicrobia* (*Akkermancia*), *Bacteroidetes* (*Rikenellaceae*, *Prevotella*, and *Rikenellaceae RC9 gut group*), and *Bifidobacterium* when treated with BPA, as compared to the control animals (Table 2) [163,166,167,169,170,171,172,173,174,175,176,177]. These discrepancies are probably due to differences in the window of exposure, exposure methods, sex of the individual, the examined host species, as well as the dose and extent of bisphenol exposure (Table 2).

Moreover, effects like changes in microbial diversity, the prevalence of *Proteobacteria*, and the increase in intestinal permeability have also been reported in fish and rabbit models (Table 2) [17,165,178,179,180].

Regarding BPA analogs, few studies have been carried out using mice, zebrafish, and human gut cultures as model systems to examine their effects on gut microbiota. Results showed that in perinatally treated mice, BPS had the most prominent effect on microbiota diversity as compared to TBBPA [18]. Early life exposure to BPS or TBBPA led to a downregulation of most species within the *Firmicutes* phylum, while less abundant taxa in the *Bacteroidetes phylum* showed consistent regulation by these endocrine disruptors. Notably, *S24-7*, the most abundant taxon in the adult fecal microbiome from the *Bacteroidetes* phylum, was significantly upregulated following early life exposure to BPS (Table 2). BPS and TBBPA perinatal animal exposure presented specific changes in some microbiota, but the most distinct microbial biomarkers were *Rikenellaceae* for TBBPA and *Lactobacillus* for BPS (Table 2) [18]. The direct exposure of female mice to BPS also induced gut microbiota dysbiosis, presenting a significant reduction in *Acidobacteria* and a large increase in *Actinobacteria* in comparison with the control group (Table 2) [163]. While *Bacillales* were significantly induced, *Sphingomonadales* and *Caldilineales* were markedly reduced in BPS-treated female mice (Table 2) [163]. Male mice exposed to bisphenol P (BPP) also presented decreased microbiota diversity and dysbiosis. Animals exposed to BPP exhibited a higher proportion of *Firmicutes*, a lower proportion of *Bacteroidetes*, and an increased *Firmicutes/Bacteroidetes* ratio compared to the control animals (Table 2) [181]. A similar increase in the *Firmicutes/Bacteroidetes* ratio was also observed in mice treated with BPS [163] or BPF [171]. An elevated *Firmicutes/Bacteroidetes* ratio, leading to a reduction in the total short-chain fatty acids (SCFAs), is associated with LPS-induced inflammatory chemocytokines release, metabolic endotoxemia, and an intensified risk of metabolic disorders such as obesity and type 2 diabetes mellitus [182]. In addition, *Proteobacteria* relative abundance was also significantly increased by BPP. *Proteobacteria* was linked to fecal LPS levels and included pathogenic bacteria capable of causing several illnesses. At the genus level, BPP treatment decreased the relative abundance of *Oscillospira*, *Prevotella*, *Bacteroides*, and *Lactobacillus*, while *Helicobacter* increased significantly in relative abundance [181] (Table 2). Male mice treated with BPF also presented a significant decrease in several bacteria of the *Firmicutes* phylum, such as *Lactobacillaceae*, *Streptococcus* spp., and *Ruminococcaceae UCG-014*, while others increased (*Lachnospiraceae*, *Ruminococcaceae UCG-010*, *Oscillibacter*, and *Roseburia). Bacteroidetes*, including *Butrycimonas*, *Prevotellaceae UCG-001E*, and *Alistipes*, increased, while *Prevotellaceae UCG-011* and *Prevotella 9* decreased as compared to the control group [171] (Table 2).

In zebrafish, BPS was identified as the least potent analog compared to BPA and BPF in a toxicological essay. However, it affected the microbial community at different concentrations by increasing the quantity of potentially pathogenic bacteria, including *Flavobacterium*, *Pseudomonas*, and *Stenotrophomonas*, that could cause oxidative damage and inflammatory effects (Table 2) [17,182,183]. BPAF and bisphenol B (BPB) did not appear to alter the microbial community structure [183] (Table 2). Furthermore, simplified human intestinal microbiota showed no differences after BPS exposure [19].

Altogether, experimental evidence indicates that direct or indirect exposure to bisphenols significantly alters the structure of the gut microbiota. Identifying specific features or species within the gut microbiota may help establish potential biomarkers for assessing the risks associated with bisphenol exposure.

## 8. Gut Microbiota Regulates Intestinal Permeability

Changes in microbiota diversity have been associated with increased intestinal permeability. The intestinal barrier consists of (1) a TJ complex that connects adjacent intestinal epithelial cells (IECs) at their apical surface, forming a polarized monolayer with distinct apical and basolateral domains; (2) a mucus layer covering the surface of IECs; and (3) the intestinal microbiota [184]. Proper operation of the intestinal barrier function is essential to ensure selective permeability of the intestine. Disruption of the barrier and permeability functions has severe consequences, including bacterial translocation to the intestine, which may lead to immune activation and inflammation, often involved in many intestinal diseases, including celiac disease, colorectal cancer, inflammatory bowel disease, and irritable bowel syndrome [185]. Disruption of intestinal flora homeostasis can alter intestinal permeability. An overgrowth of *Proteobacteria* may modify the structure and conformation of the intestinal TJs, resulting in increased intestinal permeability and absorption of lipopolysaccharides (LPS), which then enter the bloodstream (Table 2) [164,165,173,186]. Therefore, an excess of *Proteobacteria* has been associated with the onset of intestinal inflammation and intestinal diseases [187,188].

In contrast, *Akkermansia* is recognized for its ability to augment the thickness of the intestinal mucus. This mucus consists of mucins (mucin 2 being the most abundant mucin covering the intestinal epithelial cells), digestive enzymes, antimicrobial peptides, and immunoglobulins, and it improves gut barrier function, resulting in a beneficial immune response [166,189]. A decrease in mucin 2 mRNA and protein has been reported in the gut of BPA-treated animals [166,167]. In addition, dietary intake of BPA significantly reduced the number of colonic goblet cells, which produce and secrete the mucus [166]. Therefore, in animals exposed to dietary BPA, the reduced number of goblet cells and the reduced expression of mucin 2 in the colonic epithelium suggest damage to the intestinal chemical barrier.

*Ruminococcaceae*, *Parabacteroides*, *Alloprevotella*, *Allobaculum*, *Ruminiclostridium 9*, *Clostridium*, *Odoribacter* spp., and *Oscillospira* spp., which are important producers of SCFAs, are significantly altered by exposure to bisphenols [18,165,167,170,172,173,177,188,190,191,192]. SCFAs are the final products of food fermentation by the intestinal microbiota, with acetic, propionic, and butyric acid accounting for 95% of SCFAs in the human gut [193,194]. Several studies have shown that bisphenol-induced alteration of the intestinal microbiota is accompanied by a decrease in the concentration of SCFAs (acetic acid, propionic acid, butyric acid, isobutyric acid, and caproic acid) in the gut (Table 2) [18,165,167,172,173].

These SCFAs provide energy for the host and play a role in various metabolic processes in the body, including energy metabolism, the inflammatory response, adipose tissue development, and liver metabolism [193,195,196]. SCFAs help in the maintenance of intestinal homeostasis by stimulating mucus production, inducing epithelial cells to synthesize antimicrobial peptides (such as β-defensins and REG3γ), increasing the expression of intestinal TJ proteins, and preserving the integrity of the intestinal epithelial barrier. A healthy gut epithelial barrier prevents bacterial and LPS translocation into the bloodstream. Among the various SCFAs, butyric acid is considered the most prominent, as several studies have shown that it effectively regulates the apoptotic pathway in cells, prevents colon cancer, reduces bacterial translocation and the inflammatory response, and enhances intestinal barrier function by boosting TJ protein expression, thereby improving gut defense barriers [197]. Accordingly, a decrease in the expression of TJ mRNA and proteins (occludin, claudin-1, claudin-4, and ZO-1) has been reported in the gut of animals exposed to bisphenol (Table 2) [165,166,167,173,179,181]. The TJs are crucial components of the intestinal physical barrier, as they regulate the selective permeability of the intestinal epithelium by controlling paracellular pathways [198]. The function of TJs is to permit the passage of ions and small soluble molecules while blocking toxic substances and microorganisms, thereby playing a vital role in maintaining the intestinal barrier [199]. Occludin and claudins are integral TJ proteins that play a role in regulating epithelial permeability and are key components of the filamentous structures of TJs detected by freeze–fracture microscopy [200]. ZO-1 is a peripheral membrane protein of TJs, serving as a scaffold that brings together structurally diverse yet functionally related proteins in the near vicinity of the TJs. Thus, ZO-1 connects occludin and the claudins to the actin cytoskeleton. The levels of occludin and ZO-1 in tissue are inversely associated with its permeability and directly related to the maintenance of intestinal epithelium integrity [201]. Bisphenols markedly decreased the expression of ZO-1, claudin-1, claudin-4, and occludin and significantly disturbed the TJs amongst intestinal epithelial cells, affecting the colonic epithelial physical barrier function. Moreover, it has been shown that exposure of human colon mucosal epithelial cells (NCM460) to BPS and BPF increased the permeability of intestinal mucosa by down-regulating the expression of TJ proteins claudin-1 and ZO-1 [202,203]. In addition, the concentrations of serum LPS, endotoxins, zonulin, DAO, and D-lactate (biomarkers of gut permeability) [204], were also increased along with the breakdown of TJs (Table 2) [164,165,166,172,198], indicating that bisphenol exposure impaired the intestinal barrier function, increased the intestinal permeability, and enhanced serum LPS levels.

There are several important receptors in the cells of the intestinal barrier that monitor the environment of the intestinal lumen. These pattern-recognition receptors (PRRs) are present in various cell types in diverse proportions, depending on the cell’s function, and they can detect a broad range of pathogen-associated molecular patterns (PAMPs), including viral and bacterial components. PRRs encompass several super-families like Toll-like receptors (TLRs), NOD-like receptors (NLRs), RIG-I-like receptors (RLRs), and C-type lectin receptors (CLRs). TLRs are capable of detecting a wide array of molecules, including those from fungi, viruses, bacteria, and protozoa. In mammals, 13 distinct TLRs have been described (human: TLR1-11; mouse: TLR1-9, TLR11-13), with only minor functional modifications noted between humans and mice [205]. TLRs are transmembrane proteins featuring an extracellular domain with leucine-rich repeats and an intracellular domain similar to the IL-1R, known as the Toll/IL-1R (TIR) domain. When activated, the TIR domain binds to a homologous domain in the protein Myeloid Differentiation Primary Response 88 (MyD88), initiating a signaling cascade that activates the nuclear factor-κB (NF-κB). NFκB-dependent gene transcription controls a range of genes, including those involved in the immune response, such as cytokines and chemokines, as well as cell adhesion molecules, growth factors and their receptors, and apoptosis-related genes [206]. TLR expressly differentiate between self- and microbial non-self by recognizing widely conserved molecular patterns. They are crucial in microbial recognition, the regulation of adaptive immune responses, and the activation of antimicrobial effector pathways, which contribute to the effective elimination of pathogens that threaten host health. Particular TLRs are expressed along the apical and basolateral domains of the IEC membranes, which has led researchers to speculate that microbial ligands regulate TJ proteins through their contact with TLRs. Indeed, TLRs play a role in maintaining IEC homeostasis by controlling the host’s responses to intestinal microbes. Some of the receptors that have been involved in this function are TLR2 and TLR4. In mice, TLR expression changes significantly along the length of the intestinal tract. Colonic IECs show a high expression of TLR2 and TLR4, whereas small intestinal IECs have very low quantities of these receptors [205]. This difference may be due to the fact that, under healthy conditions, the small intestine harbors a less concentrated microbiota compared to the colon [207]. Healthy human colon tissues contain low levels of TLR2 and TLR4 in the IECs [205]. Where detected, TLR2 and TLR4 are located on the apical surface of the epithelial cells. This apical localization allows TLRs to be stimulated by substances in the intestinal lumen, including both commensal bacteria and harmful pathogens [207]. TLR2 and TLR4 recognize PAMPs from Gram-positive and Gram-negative bacteria, respectively. For effective LPS-induced signaling, TLR4 must associate with MD-2, a secreted glycoprotein, and CD14 [205]. Another TLR family member, TLR2, is activated by various molecules, including peptidoglycan, zymosan, and bacterial lipopeptides, to trigger innate immune responses. TLR2 forms a functional heterodimeric complex with either TLR1 or TLR6 on the surface of enterocyte and immune cells [208]. The TLR1 and TLR6 subunits are crucial for recognizing microbe-associated molecular patterns (MAMPs). Bacterial cell wall components, such as triacyl and diacyl lipopeptides, are detected by TLR2/TLR1 and TLR2/TLR6 heterodimers, respectively [208]. Stimulation of two different IEC lines (HT-29, Caco-2) with TLR2 ligand Pam3CysSK4 (which is a synthetic bacterial lipopeptide) resulted in significant activation (phosphorylation, enzymatic activity, and translocation) of specific PKC isoforms (PKCα and PKCδ). The activation of TLR2 by ligands significantly increased transepithelial resistance in IECs, an effect that was blocked by pretreatment with PKC-selective antagonists. This response was associated with the tightening and sealing of apical TJ-associated ZO-1, mediated by PKC in response to TLR2 ligands, while no noticeable morphological changes were observed in occludin, claudin-1, or the actin cytoskeleton [209]. In addition, specific *Lactobacillus* species mitigated barrier disruption by upregulating TJ proteins. *L. acidophilus LA1* and *L. plantarum MB452* were shown to augment occludin protein expression in vivo and in vitro models, respectively, through stimulation of TLR2 [208,210]. In addition, *L. plantarum* increased occludin protein expression and induced apical relocalization of ZO-1 and occludin.

Interestingly, TLR activation does not always lead to improved barrier function. LPSs, key components of the Gram-negative bacterial cell wall, play a crucial role in driving intestinal inflammatory responses associated with inflammatory bowel disease. In vitro and in vivo studies have demonstrated that physiologically relevant concentrations of LPS increase intestinal epithelial TJs permeability through the TLR4/MyD88 signal-transduction pathway, leading to up-regulation of myosin light chain kinase (MLCK) expression and activity. MLCK facilitates the opening of TJs by contracting the perijunctional ring of actin–myosin filaments. This process generates contractile tension and causes centripetal retraction of the perijunctional plasma membrane and the TJ complex, ultimately resulting in the functional opening of the TJ barrier [211].

Therefore, the changes in gut microbiota diversity produced by bisphenols have been related to the decrease in the expression of TJ proteins in the gut [166,167], leading to increased intestinal permeability, presence of endotoxins in serum and imbalance in the immune system by gut and liver inflammation [167,168].

These results can guide future research and policies regarding the safety of bisphenols, emphasizing the necessity for stricter regulations and limitations on the use of these compounds in consumer products.

## 9. Uterine Microbiota May Regulate Implantation Through Toll-like-Receptors (TLRs)-Tight Junctions (TJs)

As previously mentioned, changes in the composition of resident microbiota present in the uterus can affect implantation success. There are no studies on the impact of bisphenols on the EM, but as it has been reviewed, bisphenols influence the intestinal microbiota species. These changes produce alterations in the intestine permeability, which is partly regulated through TLR and TJs.

The genital epithelial cells express a wide range of PRRs that promote the ability to recognize and differentially respond to various pathogens. The PRRs found in the FRT include TLRs and NOD-like receptors, which play crucial roles in defending against pathogenic invasion, supporting tissue adaptation, and ensuring successful reproduction. In human endometrial tissues, the expression of TLR1-10 has been reported, and it has been proposed that these receptors are involved in microbial recognition and immune defense within the reproductive tract [212]. Studies have demonstrated that TLR mRNA and protein levels vary through the menstrual cycle [212,213,214,215,216,217]. Specifically, the expression of TLRs 1–10 in endometrial cells is low during the proliferative phase and increases during the secretory phase. Additionally, research indicates that the activation of TLRs by their specific ligands around the time of embryo implantation negatively impacts implantation outcomes both in vivo [215] and in vitro [216,217]. Most of these studies evaluate the immune response produced by the activation of TLRs. For instance, it has been reported in mice that the activation of TLR2 and 2/6 reduced embryo implantation chances and increased levels of proinflammatory cytokines, including the interleukin (IL)-1β and monocyte chemotactic protein (MCP)-1, in uterine horn flushing on the preimplantation day, along with increased IL-1 receptor antagonist on the implantation day [215]. In vitro, the stimulation of TLR3 in endometrial cells resulted in a decreased proportion of trophoblasts adhering to endometrial cells and a significant reduction in CD98 expression. These alterations were shown to be regulated via MYD88-MAPK pathways [216]. A similar decrease in the proportion of trophoblasts adhered to endometrial cells was reported for TLR5, with an increased expression of IL-8 and MCP-1 [217]. The immune response during implantation and early pregnancy has been widely studied, and providing a detailed review lies outside the scope of this work. Recent reviews address this subject [71,218,219].

However, there is scarce information about the effects of microbiota-activated TLRs on the regulation of TJ proteins in the endometrium during implantation. Some in vivo and in vitro studies with human endometrial epithelial cell lines have reported the effect of diverse microorganisms on the expression of TJ proteins. For example, in ECC-1 cells, a human endometrial epithelial cell line originating from endometrial adenocarcinoma, live *Chlamydia trachomatis* (*C. trachomatis*) but not heat-killed forms, has been shown to reduce transepithelial resistance (TER), indicating compromised TJ barrier integrity, and decrease the expression of TJ genes, including occludin, claudin-2, claudin-3, and ZO-1 [220]. This indicates that an active infection with live bacteria is necessary to compromise the integrity of the epithelial cell barrier, as exposure to factors on the *C. trachomatis* surface alone is insufficient. Interestingly, live *C. trachomatis* did not affect the TER of polarized rat endometrial epithelial cells [221], suggesting species-specific variations in endometrial responses to infection. *C. trachomatis*, the most prevalent sexually transmitted bacteria pathogen worldwide [222], has been associated with miscarriage, stillbirth, and preterm premature rupture of membranes [223,224,225]. Similarly, *Neisseria gonorrhea (N. gonorrhea)*, a Gram-negative bacterium and the second most common sexually transmitted bacterial pathogen globally, can ascend through the cervix, producing pelvic inflammatory disease linked to tubal infertility, stillbirth, and preterm birth [226]. Infection of the polarized human endometrial epithelial cell line HEC-1B with live but not gentamicin-killed *N. gonorrhea* disrupted cell junctional complexes, redistributing ZO-1, occludin, E-cadherin, and β-catenin from the apical surface to the cytoplasm without disturbing total protein levels [227]. Similarly, in polarized Ishikawa cells, *N. gonorrhea* provoked the rearrangement of E-cadherin and β-catenin from the apical membrane to the cytoplasm but did not alter the expression or localization of the TJ proteins occludin and ZO-1 [228]. *N. gonorrhea* infection enhanced TLR2 and TLR4 expression and p65 NF-κB levels and reduced nuclear factor erythroid 2-related factor 2 (Nrf2), driving the inflammatory response [229]. *Staphylococcus aureus* (*S. aureus*), a Gram-positive bacterium commonly part of the normal human microbiota, can act as an opportunistic pathogen associated with infertility in both sexes [230,231]. Elevated endometrial levels of *S. aureus* have also been linked to poor IVF outcomes [232]. In mice uterus, *S. aureus* reduced the expression of the TJ proteins ZO-1, occludin, and claudin-3 [233] while inducing uterine structural disruption, luminal dilation, and fluid accumulation [234]. In both rats and mice, *S. aureus* increased Tlr2 expression and activated IκB and p65 NF-κB phosphorylation [235,236]. Additionally, *S. aureus* elevated uterine Tlr4 expression [237] and downstream TLR signaling mediators, including MyD88, IRAK1/4, and TRAF6 [236]. In vitro studies further showed that *S. aureus* increased TLR2 levels and enhanced IκB and p65 NF-κB phosphorylation in mouse epithelial cells [238].

## 10. Tight Junctions (TJs) Participation in Embryo Implantation

In the uterus, TJs join the uterine epithelial cells (UECs) lining the lumen and regulate the passage of ions and molecules through the paracellular path. Protein composition of TJs present in the luminal UECs changes during the different days of the menstrual/estrous cycle and of pregnancy, which suggests that the expression of TJ proteins participates in providing an adequate environment for successful fertilization and implantation [239,240,241,242,243]. Indeed, TJs are implicated in the transformation of the plasma membrane that renders UECs receptive to the attachment of trophoblastic cells and control the composition and volume of the luminal fluid, which serves multiple purposes, such as aiding in the maturation of the ovum and spermatozoa and providing nutrients and signaling molecules for the implanting blastocyst [244]. The movement of luminal fluid is regulated in the UECs by TJs. While these early embryo–maternal interactions cannot be studied directly in humans, extensive research has been carried out in various animal models. On gestational day (GD) 1, the edema observed in the rat endometrium relies on the movement of fluid from the stroma into the uterine lumen via the paracellular pathway, facilitated by leaky TJs consisting of parallel strands located at the apical region of the plasma membrane [245,246]. During this GD, ZO-1 and claudin-1 and -3 were detected in the lateral membrane, whereas claudin-7, associated with cell-to-extracellular matrix attachment [247], was also observed [243,244,246,247,248,249,250,251]. Claudin-1 and -3 establish heterotypical interactions with each other [252], and claudin-1 produces linear, non-branching arrays of strands [250] similar to those observed during this GD.

During blastocyst implantation, marked by the irreversible adhesion of the blastocyst to the luminal epithelium on GD 6 in the rat, the TJ network expands threefold in depth along the lateral plasma membrane. It also develops additional branches and interconnections with adjacent strands, as revealed by freeze–fracture microscopy [245,246,251]. On this GD, ZO-1 was predominantly localized in the upper third of the lateral plasma membrane, claudin-1 in the lower half, claudin-3 throughout the lateral membrane, and claudin-7 in the lower half [243]. For the first time during this GD, claudin-4 was detected at the basolateral membrane of the UECs [243,249]. Additionally, increased claudin-4 mRNA expression has been described during the implantation window in humans [253,254], indicating a potential functional role in this process. Claudin-4 overexpression in cultured cells is known to increase the complexity of the TJ strand network [255], suggesting that it enhances the number of branches and interconnections observed by freeze–fracture microscopy on GD 6. Meanwhile, claudin-7 expression decreased, and its localization shifted from the basolateral region seen on GD 1 to the lower half of the basolateral membrane [246,247]. Claudin-4 reduces paracellular Na^+^ permeability [255], whereas claudin-7 acts as a paracellular Na^+^ channel and a Cl^−^ barrier [256]. At the implantation stage, epithelial Na^+^ channels (ENaC) are induced in rat UECs, reducing luminal Na^+^ levels [257,258]. The concurrent presence of ENaC and claudin-4, along with the absence of claudin-7 from the TJ region, ensures efficient transcellular Na^+^ movement from the apical to the basal epithelial surface without paracellular backflow. This Na^+^ transport, combined with water reabsorption from the luminal fluid via aquaporin 5 [259], enables the blastocyst to closely align with the luminal epithelium, facilitating surface contact [258,260]. The reduced expression of claudin-7 on GD 6 may also weaken cell-to-extracellular matrix adhesions, contributing to the loosely adhered epithelial monolayer characteristic of implantation day [261]. Claudin-7 forms a stable protein complex with claudin-1 and integrin α2, suggesting that it stimulates cell–matrix adhesion by stabilizing integrin α2 proteins, which mediate connection with extracellular matrix components [247].

In rodents, during the blastocyst’s penetration of the UECs into the stromal layer (GD 7), the presence of ZO-1, occludin and claudin-1, -3, and -4 has been detected in stromal cells [243,262,263]. These proteins may create a barrier within stromal cells to shield the blastocysts from maternal immunoglobulins.

## 11. Impact of Bisphenols on Uterine Epithelial Cells (UECs) Tight Junctions (TJs)

There is scarce information on the effects of bisphenols on UEC TJ proteins during pregnancy. Rats treated with BPA during the perinatal period were mated at 3 months of age. In these animals, BPA treatment did not induce changes in the ovulation rate, but it reduced the implantation rate [243].

The most important alteration observed in the expression of UEC TJ proteins was the presence of claudin-4 from GD 1, whereas, in the control group, this claudin was only expressed at a high level on GD 6. The premature expression of claudin-4 could reduce paracellular Na^+^ permeability in UEC, potentially altering the composition of the luminal fluid, which could hinder the implantation process [242].

BPA exposure also induced an alteration in the localization of ZO-1 on GD3. In the control group, ZO-1 protein was highly expressed along the basolateral membrane in UECs, whereas in BPA-treated groups, its expression was reduced and confined to the uppermost portion of the lateral membrane [242]. Given that ZO-1 serves as a scaffold for claudin polymerization [264], BPA treatment may hinder the increased depth of TJs typically observed on GD 3 [244,245].

Claudin-7 also showed important differences between the BPA-treated group and control rats. In the control group, this protein was located in the basolateral membrane of UECs on GD 1 and shifted to the lower half of the lateral plasma membrane by GD 6 [242]. In the BPA-treated group, claudin-7 expression decreased in UECs on GD 1, with the protein localized to the lower half of the lateral plasma membrane. This change overlapped with a rise in claudin-4 protein expression, supporting the opinion that BPA treatment promotes the formation of a cation-impermeable TJ on GD 1. Furthermore, as claudin-7 enhances cell–extracellular matrix adhesion [246], its reduced expression during the non-receptive phase may hamper the conservation of an intact uterine epithelial barrier.

On GD 7, perinatal BPA treatment produced the loss of claudin-3 and -4 in stromal cells. The absence of these proteins may disrupt the formation of a barrier between stromal cells, which normally prevents maternal immunoglobulins from reaching the embryo [242].

In conclusion, BPA exposure creates an intrauterine environment that hinders successful embryo implantation during early pregnancy.

## 12. Conclusions and Future Directions

The dogma that the uterus was a sterile environment has come to an end. The female upper reproductive tract has its own microbiota, but the “baseline” or “core” microbiome of the healthy endometrium has not been completely established due to limitations in the studies, such as the challenging collection of uncontaminated uterine samples, free from vaginal or cervical bacteria and bacterial DNA found in the air and laboratory reagents and equipment. Other limiting factors are fluctuations due to hormonal changes within the menstrual cycle, the lack of ethnic diversity, differences in socioeconomic status, lifestyle, and environmental factors. However, it has become clear that the predominant phyla identified in the endometrium of healthy women are *Firmicutes* (mainly *Lactobacillus* spp.), *Bacteroidetes* (mainly *Flavobacterium* spp., *Bacteroides* spp., *Prevotella* spp.), *Proteobacteria* (mainly *Pseudomonas* spp. and *Acinetobacter* spp.), and *Actinobacteria* (mainly *Gardnerella* spp., *Bifidobacterium* spp.) [24,26,48,59,60,61,62]. It has been proposed that several gynecological diseases, such as chronic endometritis, endometriosis, adenomyosis, hysteromyoma, endometrial hyperplasia, cancer, and infertility, may be related to dysbiosis of the microbial community present in the endometrium. Studies in women undergoing IVF have suggested that a NLDM (<90% *Lactobacilli* with >10% of other bacteria) was associated with infertility, with significantly decreased implantations, pregnancies, and live births. However, some patients achieved pregnancies despite having 0% *Lactobacillus*. Furthermore, other studies proposed that the primary factor affecting fertility may be the occurrence of pathogens in the uterine cavity rather than the necessity of a specific commensal taxon [24,38,72,96,102,104,106,108,109,110,111,112,113,114,115]. Therefore, it has been postulated that the primary role of *Lactobacillus* spp. in reproduction is to prevent the establishment of pathogenic bacteria in the uterine cavity [72]. These data suggest that the EM may be regarded as an emerging factor contributing to implantation failure and/or pregnancy loss. As a result, microbial interventions with antibiotic therapy, followed by the combination of prebiotics and/or probiotics, have been used to change the NLDM into LDM prior to subsequent conception attempts in order to improve infertility treatment outcomes and IVF success [74].

Bisphenols are one of the environmental factors that may contribute to EM dysbiosis, similar to what has been reported in the gut microbiota. In the human gut, *Firmicutes* and *Bacteroidetes* are the dominant phyla (representing at least 90%), followed by *Proteobacteria* and *Spirochaetae* [162]. The gut microbiota produces SCFAs that contribute to the maintenance of intestinal homeostasis by stimulating mucus production, inducing epithelial cells to synthesize antimicrobial peptides, increasing the expression of intestinal TJ proteins, and preserving the integrity of the intestinal epithelial barrier. In several animal models, bisphenol exposure reduced microbiota diversity and significantly altered the structure of the gut microbiota. This microbiota dysbiosis is detected by TLRs, which induce changes in the expression/localization of TJ proteins, the same that regulate the selective permeability of the intestinal epithelium by controlling paracellular pathways [198]. TJ proteins are composed, among others, of occludin and claudins, which are integral TJ proteins, and ZO proteins that connect occludin and claudins to the actin cytoskeleton. Bisphenols markedly decreased the expression of ZO-1, claudin-1, claudin-4, and occludin and significantly disturbed the TJs amongst intestinal epithelial cells, affecting the colonic epithelial physical barrier function, which results in the absorption of LPS, endotoxins, zonulin, DAO, and D-lactate, which then enter into the bloodstream. In the endometrium, TJ proteins are important during embryo implantation, and their expression and localization are affected by exposure to bisphenols. TJs are implicated in the transformation of the plasma membrane that renders UECs receptive to the attachment of trophoblastic cells and control the composition and volume of the luminal fluid, which serves multiple purposes, such as participation in the maturation of the ovum and spermatozoa and providing nutrients and signaling molecules for the implanting blastocyst [244]. BPA exposure, by altering the expression/localization of TJ proteins during early pregnancy, creates an intrauterine environment that hinders successful embryo implantation. The knowledge that the microbiota may regulate TJs in the endometrium via TLRs, as in the gut, opens new treatment options for RIF patients. However, it is imperative to investigate the effect of bisphenols on the uterine microbiota and to establish their participation in gynecological diseases, including infertility, for future risk assessments regarding the effects of bisphenols on reproduction.

## Figures and Tables

**Table 2:** Changes in the abundance of intestinal bacteria on different model systems treated with bisphenols.

Ref.	Model System	BP Dose	AgeExp	Experimental Time Span	Age Coll	Change in the Abundance of Bacterial Taxon (Phylum, Order, Family, Genus, or Species)	Intestinal Permeability (Serum LPS) and SCFAs	TJs and Mucin 2
[13]	Male CD-1 Mice *n* = 4	BPA in water 120 μg/mL	3 weeks old	10 weeks	13 weeks old	**↑** *Proteobacteria: Epsilonproteobacteria*, *Helicobacter ganmani.* **↓** *Firmicutes*: *Clostridium viride*, *Coprococcus comes*, *Eubacterium dolichum*, *Lactobacillus intestinalis*, *Tenericutes.*		
[17]	Zebrafish (*Danio rerio*) larva	BPA, BPAF, BPB, BPF, BPS (0.2 to 45 μM)	1 DPF	10 days	10 DPF	**BPS:** **↑** *Cryomorphaceae* **↓** *Chitinimonas*, *Leptothrix*, *Neisseriaceae*, *Pseudomonas*, *Rheinheimera.* **BPA and BPF**: **↑** *Chromatiaceae*, *Leptothrix*, *Pseudomonas*, *Rheinheimera* **↓** *Chitinimonas*, *Neisseriaceae.* BPAF and BPB did not disrupt microbial community structure.		
[18]	Pregnant CD-1 mouse	TBBPA 0.2 mg/Kg/d or BPS 0.2 mg/Kg/d	Adult dams	GD 8 to PND 21	F1: 20-week-old adult male pups	**TBBPA and BPS**: **↓** *Bacteroidetes uniformis*, *Clostridiales*, *Lachnospiraceae*, *Oscillospira*, *RF39*, *Ruminococcaceae*, *Ruminococcus*, *Ruminococcus gnavus* **BPS:** **↑** *Adlercreutzia*, *Bacillus cereus*, *Gemellaceae*, *Lactobacillus*, *Lautropia*, *S24-7* **↓** *Bacteroidaceae*, *Bacteroides*, *Odoribacter*, *Rikenellaceae* **TBBPA:** **↑** *Alkaliphilus*, *Anaerotruncus*, *Bacillales*, *Bacillus*, *Bacteroides*, *Candidatus arthromitus*, *Coriobacteriaceae*, *Lactococcus garvieae*, *Lactococcus*, *Rikenellaceae*, *Streptococcus agalactiae* **↓** *Anaerostipes*, *Coprobacillus*, *Roseburia*	**BPS**: **↓** acetic acid **TBBPA:** **↑** propionic acid, succinate	
[163]	Female C57BL/6J mice	BPS 1.5 μg/d	3 weeks old	22 weeks	25 weeks old	**↑** *Actinobacteria*, *Bacillales*, *Coriobacteriales* **↓** *Acidobacteria*, *Acidobacteriales*, *Caldilineales*, *Gaiellales*, *Sphingomonadales* **↑** *Firmicutes/Bacteroidetes* ratio		
[165]	Pregnant Dutch belted rabbits *n* = 4/grp	BPA 200 μg/Kg BW/d	Adult	F0 females: GD 15 (midgestation) to PND 7	F1: 6 weeks of age	**F0 dams fecal samples:** **↑** *Acinetobacter*, *Jeotgalicoccus*, *Oscillospira* spp. **F1 fecal samples:** **↓** *Akkermansia* spp., *Odoribacter* spp.	Serum: **↑** LPS Feces: **↓** acetic and propionic acid	
[166]	Male CD-1 mice *n* = 10/grp	BPA in water 50 μg/Kg/d	3 weeks old	10 weeks	13 weeks old	Colonic mucosa **↑** *Subdoligranulum* **↓** *Eubacterium coprostanoligenes group*, *Oscillospira*, *Prevotella 1*, *Prevotella 2*, *Prevotellaceae NK3B31 group*, *Rikenellaceae RC9 gut group Ruminococcaceae NK4A214 group*, *Ruminococcaceae UCG 002*, *Ruminococcaceae UCG 010*	In plasma and colonic mucosae: **↑** DAO, endotoxins, D-lactate, zonulin	mRNA: **↓** muc2, ZO-1, occl, cldn-1 Protein: **↓** ZO-1, occl, cldn-1
[164]	Male CD-1 mice *n* = 8/grp	BPA in diet 50 μg/Kg BW/d	6 weeks old	24 weeks	30 weeks old	**↑** *Proteobacteria* **↓** *Akkermansia*, *Verrucomicrobia*	Serum: **↑** LPS, DAO, D-lactate	Protein: **↓** ZO-1, occl
[167]	Male and female C57BL/6J mice	BPA in diet 0.05, 0.5, 5, and 50 mg/Kg/d feed weight	8 weeks old	22 weeks	30 weeks old	**Male:** **↑** *Firmicutes*, *Oscillibacter*, *Rikenellaceae RC9 gut group*, *Ruminiclostridium 9*, *Ruminococcaceae NK4A214 group*, *Tyzzerella*, *Verrucomicrobia.* **↓** *Akkermansia*, *Allobaculum*, *Alloprevotella*, *Bacteroidetes*, *Christensenellaceae R 7 group*, *Marvinbryantia*, *Muribaculum*, *Parabacteroides*, *Parasutterella*, *Proteobacteria*, *Ruminococcaceae UGG 010*, *Ruminococcaceae UGG 013*, *Ruminococcus 1.* **Female:** **↑** *Bilophila*, *Desulfovibrio*, *Enterorhabdus*, *Firmicutes*, *Millionella*, *Peotococcus*, *Proteobacteria*, *RikenellaceaeRC9 gut group*, *Ruminococcaceae UGG 009.* **↓** *Bacteroides*, *Bacteroidetes*, *Muribaculum*, *Parasulterella.*	**Male:** **↓** propionic acid, caproic acid **Female:** **↓** butyrate acid	**Male** mRNA and protein: **↓** muc2, ZO-1, occl, cldn-1 **Female** mRNA and protein: **=** muc2, ZO-1, occl, cldn-1
[168]	Pregnant C3H/HeN mice *n* = 10–16/grp	BPA 50 μg/Kg BW/d	8 weeks old	F0 females: GD15 to PND 21	F1 males: PND 45 and 170	**↓** *Firmicutes*: (*Clostridium butyricum*, *Clostridium Cluster XIVa); Bifidobacterium* spp.		
[169]	Pregnant C57BL/6J mice	BPA 5 μg/Kg/d	F0 Adults	F0 dams: Gestation period (19–21 days)	F1 samples at 11, 14, and 20 weeks of age	**F1 females:** **↑** *Rikenellaceae*, **↓** *Prevotella.* **F1 males:** **↑** *Rikenellaceae*, **↓** *Lactobacillus.*		
[170]	Pregnant Sprague Dawley rats	BPA 50 μg/Kg/d	F0 15 weeks old	F0 dams: GD 6 to PND21	F1 males PND 50	**↑** *Allobaculum*, *Blautia*, *C. ruminantium*, *Lactobacillaceae*, *L. reuteri*, *Prevotella.* **↓** *Adlercreutzia*, *Oscillospira*	**↑** acetic acid, propionic acid	
[171]	C57BL/6 male mice	BPA 50 μg/kg/d and 5 mg/Kg/d BPF 50 μg/Kg/d and 5 mg/Kg/d	8 weeks old	14 days	10 weeks old	**BPA 5 mg/kg/d** **↑** *Alistipes*, *Alloprevotella*, *Anaerotruncus*, *Bacteroides*, *Bilophila*, *Butyricicoccus*, *Enterorhabdus*, *Eubacterium coprostanoligenes*, *Lachnospiraceae NK4A136*, *Oscillibacter*, *Prevotellaceae UCG-011*, *Roseburia*, *Ruminiclostridium 9*, *Ruminiclostridium*, *Ruminococcaceae NK4A214*, *Ruminococcaceae UCG-010*, *Streptococcus* **↓** *Lactobacillaceae*, *Ruminococcaceae UCG-014* **BPA 50 μg/kg/d** **↑** *Butyricicoccus*, *Butyricimonas*, *Lachnospiraceae NK4A136*, *Oscillibacter*, *Prevotellaceae NK3B31*, *Ruminococcaceae UCG-010*, *Ruminococcus 1* **↓** *Lactobacillaceae*, *Prevotella 9*, *Prevotellaceae UCG-011*, *Ruminococcaceae UCG-014*, *Streptococcus* **BPF 5 mg/kg/d** **↑** *Lachnospiraceae FCS020*, *Lachnospiraceae NK4A136*, *Oscillibacter*, *Ruminococcaceae UCG-010* **↓** *Lactobacillaceae*, *Prevotellaceae UCG-011*, *Ruminococcaceae UCG-010*, *Ruminococcaceae UCG-014* **BPF 50 μg/kg/d** **↑** *Alistipes*, *Butyricimonas*, *Lachnospiraceae NK4A136*, *Oscillibacter*, *Prevotellaceae UCG-001E*, *Roseburia*, *Ruminococcaceae UCG-010* **↓** *Lactobacillaceae*, *Prevotella 9*, *Prevotellaceae UCG-011*, *Ruminococcaceae UCG-014*, *Streptococcus* spp.		
[172]	Pregnant Hu ewes *n* = 8/grp Gut microbiota transplantation (GMT) from pregnant ewes to microflora-free mice (removed by antibiotics).	Hu ewes: BPA 5 mg/Kg/d sc injection	Hu ewes: 18.7 ± 0.6 months Mice: 7 weeks old	Hu ewes: GD 40 to GD 110. Mice: GMT for 6 weeks administration of ewe fecal supernatant and afterwards from GD 0 to GD 18	Hu ewes: GD 110 Mice: GD 18	Ewes and mice: **↑** *Firmicutes*, *Proteobacteria*, *Veillonella* **↓** *Actinobacteria*, *Bacteroides*, *Bacteroidetes*, *Bifidobacterium*, *Clostridium*, *Lactobacillus* **↑** *Firmicutes/Bacteroidetes* ratio	Colonic content: **↓** acetate, butyrate, propionate, isobutyrate, LPS	
[173]	Male C57BL/6j mice	BPA 50 mg/Kg BW/d	8 weeks old	6 weeks	14 weeks old	**↑** *Bifidobacterium*, *Faecalibaculum*, *Parasutterella* **↓** *Alloprevotella*, *Bacteroides*, *Helicobacter*, *Lactobacillus*	Feces: **↓** Acetate, propionate, butyrate Serum: **↑** LPS	Protein: **↓** ZO-1, occl
[174]	Pregnant California mouse *n* = 6/grp	BPA in diet 50 mg/Kg feed weight	F0 females 2 weeks prior to breeding F0 males from breeding	Gestation and lactation (30 days)	F0: Adults F1: PND 30	**F0 Females**: **↑** *Clostridiales*, *Mogibacteriaceae*, *Sutterella* spp. **↓** *Lactococcus* spp. **F0 males:** **↑** *Mollicutes*, *Prevotellaceae.* **↓** *Desulfovibrio* spp. **F1 Females:** **↑** *Bifidobacterium* spp., *Mogibacteriaceae*. **↓** *Oxalobacter* spp. **F1 males:** **↑** *Akkermansia* spp., *Methanobrevibacter* spp., *Sutterella* spp. **↓** *Proteobacteria*, *Desulfovibrio* spp.		
[175]	Female NOD mice *n* = 6/grp	BPA by gavage using micropipetting. 30 μg/Kg BW/d	Juvenile	PND 28 to PND 56	PND 134	**↑** *Akkermansia*, *Anaerofustis*, *Jeotgalicoccus*, *Lachnospiraceae*, *Oscillospira*, *Rhodospirillales*, *Ruminococcus*, *TA18*, *Turicibacter*, *Verrucomicrobia*, *Verrucomicrobiae* **↓** *0319 6A21*, *Acidobacteriia*, *AD3*, *EB1017*, *Ellin329*, *Gemmatimonadetes*, *Gitt GS 136*, *JG37 AG 4*, *Koribacteraceae*, *N1423WL*, *Nitrospira*, *Nitrospirae*, *OD1*, *SC I 84*, *Sinobacteraceae*		
[176]	Pregnant California mouse *Peromyscus californicus*	BPA in diet LD 5 mg/Kg feed weight UD 50 mg/Kg feed weight	F0 Adults	F0 dams: 2 weeks before mating and during gestation and lactation (30 days).	Adults	**F1 LD BPA females:** **↑** *Akkermansia muciniphila*, *Allobaculum* spp., *Blautia* spp., *Clostridiales*, *Dehalobacterium* spp., *Dorea* spp., *Enterobacteriaceae*, *Lachnospiraceae*, *Lactobacillus* spp., *[Mogibacteriaceae]*, *Oscillospira* spp., *Ruminococcaceae*, *Ruminococcus* spp. **↓** *Akkermansia* spp., *Alphaproteobacteria RF32*, *Anaerostipes* spp., *Bacteroidales f.S24-7*, *Coprococcus* spp., *Lachnospiraceae.* **F1 UD BPA females:** **↑** *Allobaculum* spp., *Lachnospiraceae*, *Lactobacillus* spp., *Rikenellaceae* **↓** *Alphaproteobacteria RF32*, *Bacteroidales f.S24-7*, *Bacteroides uniformis*, *Clostridiales*, *Coprococcus* spp., *Oscillospira* spp. **F1 LD BPA males:** **↑** *Allobaculum* spp., *Bacteroides* spp., *Blautia producto*, *Blautia* spp., *Burkholderiales*, *Clostridiaceae*, *Clostridiales*, *Coriobacteriaceae*, *Desulfovibrio* spp., *Desulfovibrionaceae*, *Dorea* spp., *[Eubacterium] dolichum*, *Lactobacillus reuteri*, *Lactobacillus* spp., *Parabacteroides distasonis*, *Parabacteroides* spp., *Peptostreptococcaceae*, *Peptostreptococcaceae*, *Porphyromonadaceae*, *Ruminococcaceae*, *Ruminococcus* spp. **↓** *Akkermansia muciniphila*, *Anaeroplasma* spp., *Bacteroidales S24-7*, *Carnobacteriaceae*, *Clostridiaceae*, *Coprococcus* spp., *Cyanobacteria c.4C0d-2 o.YS2*, *Desulfovibrionaceae*, *Lachnospiraceae*, *Lactobacillus* spp., *Odoribacter* spp., *Oscillospira* spp., *Ruminococcaceae.* **F1 UD BPA male** **↑** *Blautia* spp., *Clostridiale*, *Desulfovibrio* spp., *Desulfovibrionaceae*, *Helicobacteraceae*, *Lachnospiraceae*, *Parabacteroides distasonis*, *Parabacteroides* spp., *Porphyromonadaceae.* **↓** *Allobaculum* spp., *Bacteroidales f.S24-7*, *Lactobacillus* spp.		
[177]	Pregnant Sprague Dawley rats	BPA gavage 50 μg/Kg/d	15 weeks old	F0 dams: GD 6 to PND 21	F1 females: PND 50	**↑** *Clostridium perfringens*, *Clostridium ruminantiums*, *Prevotella.* **↓** *Firmicutes/Bacteroidetes* ratio	**↑** Acetic acid	
[178]	Male zebrafish *n* = 120/grp	BPA 2000 μg/L	Adult	5 weeks	Adult	**↓** *Acinetobacter*, *Aquabacter*, *Bacteroidetes*, *Bosea*, *Proteobacteria*, *Xanthobacter* **↑** *CKC4*, *Firmicutes*		
[179]	Male and female zebrafish (*Danio rerio*)	BPA 0, 2, and 20 μg/L	Adult	3 months	Adult	**Male and female:** **↑** *Actinobacteria* **↓** *Hyphomicrobium* **Male:** **↑** *Lawsonia*		**Female** 2 μg/L: **↑** TJP2 20 μg/L: **↓** TJP2
[180]	Channel catfish (*Ictalurus punctatus*)	BPA 500 μg/L	Juvenile	7 days	Juvenile	**↑** *Actinobacteriota*, *Firmicutes*, *Proteobacteria* **↓** *Bacteroidota*, *Clostridium*, *Fusobacteriota*		
[181]	C57BL/6 male mice *n* = 6/grp	BPP 30 or 3000 μg/Kg/d	5 weeks old	5 weeks	10 weeks old	**↑** *Bacteroidetes*, *Firmicutes*, *Helicobacter*, *Proteobacteria* **↓** *Bacteroides, Lactobacillus, Oscillospira, Prevotella* **↑** *Firmicutes/Bacteroidetes* ratio	Serum: **↑** LPS	mRNA: **↓** ZO-1, occl Protein: **↓** ZO-1, occl, cldn-4
[182]	Adult zebrafish	BPF 0.5, 5, and 50 μg/L	Embryonic stage	180 days	Adult	**↓** *Erysipelotrichaceae*, *Gemmobacter*, *Rhodobacteraceae*		
[183]	Zebrafish (AB strain, *Danio rerio*)	BPF, BPS and BPS+BPF 1, 10, 100, 1000 μg/L	5 months old	14 days	5.5 months old	**BPF** **↑** *Flavobacterium*, *Fusobacteria* **↓** *Bacteroidetes* **BPS** **↑** *Actinobacteria*, *Flavobacterium*, *Proteobacteria*, *Pseudomonas* **↓** *Bacteroidetes*, *Cetobacterium* **↑↓** *Fusobacteria*, depending on dose **BPS+BPF** **↑** *Acinetobacter*, *Proteobacteria*, *Pseudomonas*, *Stenotrophomonas* **↓** *Bacteroidetes*, *Cetobacterium* **↑↓** *Fusobacteria*, depending on dose		

AgeExp, age of the animal at the beginning of exposure; Age coll, age of the animal at the time of sample collection; BPA, bisphenol A; BPAF, bisphenol AF; BPB, bisphenol B; BPF, bisphenol F; BPS, bisphenol S; BW, body weight; d, day; cldn, claudin; DAO, diamine peroxidase; DPF, days post fertilization; GD, gestational day; grp, group; LD, low dose; LPS, lipopolysaccharides; muc2, mucin 2; occl, occludin; PND, post-natal day; SCFAs, short-chain fatty acids; TBBPA, tetrabromobisphenol; UD, upper dose; **↑**, increase; **↓**, decrease; and **=**, no change.

## Data Availability

No new data was created.

## Abbreviations

ART, assisted reproductive technology; BPA, bisphenol A; BPAF, bisphenol AF; BPF, bisphenol F; BPS, bisphenol S; EDs, endocrine disruptors; EM, endometrial microbiota; FET, frozen embryo transfer; FRT, female reproductive tract; GD, gestational day; IVF, in vitro fertilization; *L.*, *Lactobacillus;* LDM, *Lactobacillus*-dominated endometrium microbiota; LPS, lipopolysaccharides; NLDM, non-*Lactobacillus*-dominated endometrium microbiota; PCOS, polycystic ovary syndrome; RIF, recurrent implantation failure; RPL, repeated pregnancy loss; SCFAs, short-chain fatty acids; TBBPA, tetrabromobisphenol A; TJs, tight junctions; TLRs, Toll-like receptors; UECs, uterine epithelial cells.
